# Stiffness of HIV‐1 Mimicking Polymer Nanoparticles Modulates Ganglioside‐Mediated Cellular Uptake and Trafficking

**DOI:** 10.1002/advs.202000649

**Published:** 2020-07-29

**Authors:** Behnaz Eshaghi, Nourin Alsharif, Xingda An, Hisashi Akiyama, Keith A. Brown, Suryaram Gummuluru, Björn M. Reinhard

**Affiliations:** ^1^ Department of Chemistry and The Photonics Center Boston University Boston MA 02215 USA; ^2^ Department of Mechanical Engineering and The Photonics Center Boston University Boston MA 02215 USA; ^3^ Department of Microbiology Boston University School of Medicine Boston MA 02118 USA

**Keywords:** biomimetic nanoparticles, endocytosis, lectins, lipid‐wrapped nanoparticles, siglec‐1

## Abstract

The monosialodihexosylganglioside, GM3, and its binding to CD169 (Siglec‐1) have been indicated as key factors in the glycoprotein‐independent sequestration of the human immunodeficiency virus‐1 (HIV‐1) in virus‐containing compartments (VCCs) in myeloid cells. Here, lipid‐wrapped polymer nanoparticles (NPs) are applied as a virus‐mimicking model to characterize the effect of core stiffness on NP uptake and intracellular fate triggered by GM3‐CD169 binding in macrophages. GM3‐functionalized lipid‐wrapped NPs are assembled with poly(lactic‐*co*‐glycolic) acid (PLGA) as well as with low and high molecular weight polylactic acid (PLA^lMW^ and PLA^hMW^) cores. The NPs have an average diameter of 146 ± 17 nm and comparable surface properties defined by the self‐assembled lipid layer. Due to differences in the glass transition temperature, the Young's modulus (*E*) differs substantially under physiological conditions between PLGA (*E*_PLGA_ = 60 ± 32 MPa), PLA^lMW^ (*E*_PLA_^lMW^ = 86 ± 25 MPa), and PLA^hMW^ (*E*_PLA_^hMW^ = 1.41 ± 0.67 GPa) NPs. Only the stiff GM3‐presenting PLA^hMW^ NPs but not the softer PLGA or PLA^lMW^ NPs avoid a lysosomal pathway and localize in tetraspanin (CD9)‐positive compartments that resemble VCCs. These observations suggest that GM3‐CD169‐induced sequestration of NPs in nonlysosomal compartments is not entirely determined by ligand–receptor interactions but also depends on core stiffness.

## Graphical abstract

Human immunodeficiency virus‐1 mimicking monosialodihexosylganglioside‐presenting polymeric nanoparticles with different core stiffnesses are generated, and their intracellular fate in CD169‐expressing macrophages is monitored. It is found that ligand and core stiffness together determine the intracellular fate of the lipid‐wrapped nanoparticles. These findings provide new insights into the importance of the mechanical properties of the virus particle for its function in infection.

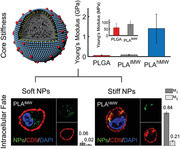

## 1 Introduction

Virus‐encoded glycoproteins have important roles in the life cycle of a virus, but it is becoming increasingly clear that in the case of enveloped virus particles, glycoprotein‐mediated receptor binding and entry can be augmented by recognition and binding of lipids incorporated within virus particle membrane by virus attachment factors, such as CD169 (Sialoadhesin or Siglec‐1).^[^ ^1^ ^]^ Human immunodeficiency virus‐1 (HIV‐1) utilizes glycoprotein‐independent, monosialodihexosylganglioside (GM3)‐dependent binding to CD169 during transinfection, a mechanism of infection in which macrophages and myeloid dendritic cells transfer captured virus particles to CD4^+^ T cells. This lectin promotes not only the binding of the virus to CD169‐expressing cells but also triggers a sequestration of virus particles in nonlysosomal compartments in dendritic cells and macrophages that offers protection from immune surveillance.^[^ ^1^, ^2^ ^]^ The unique capabilities of HIV‐1 and other viruses to target specific cells and parasitize cellular mechanisms to achieve immune evasion and establish efficient infection and replication has stimulated great interest in biomimetic artificial virus nanoparticles (NPs) that can imitate viral behavior for applications in nanomedicine and nanopharmacology without the risk of increased immunogenicity.^[^ ^3^ ^]^ We have previously shown that GM3‐functionalized gold NPs recapitulate key aspects of CD169‐dependent HIV‐1 uptake and trafficking in dendritic cells and macrophages.^[^ ^4^ ^]^

The successful reverse engineering of the intracellular fate of HIV‐1 particles with membrane‐wrapped gold NPs suggests that lipid‐mediated cell binding plays an important role in the early stages of viral infection. Intriguingly, our previously published studies have indicated that GM3 loaded onto membrane‐wrapped gold NPs have a higher propensity for sequestration into nonlysosomal compartments than liposomes with the same size and membrane composition in CD169‐expressing macrophages.^[^ ^4d^ ^]^ These observations are consistent with the model that interactions between ligand‐functionalized NPs (endogenous or artificial) and cells are in general not exclusively determined by chemical recognition of the ligand but are also dependent on the physicochemical properties of the NPs (size, surface charge, shape, and others).^[^ ^5^ ^]^ These additional NP‐specific parameters can lead to differences in uptake even if both NPs carry the same ligand. Variations in size and shape, for instance, can result in different uptake rates and for large morphological differences even trigger different uptake mechanisms.^[^ ^6^ ^]^ NP stiffness is another important physical parameter that has recently attracted significant theoretical^[^ ^7^ ^]^ and experimental^[^ ^8^ ^]^ attention for its role in NP uptake. Prior work revealed that differences in core stiffness impact both the energetics and kinetics of plasma membrane wrapping around NPs and that these changes have important implications for the efficiency of NP internalization. NP stiffness was shown to affect not only the mechanism of NP internalization^[^ ^8b^ ^]^ but also intracellular trafficking.^[^ ^8e^ ^]^ The reported stiffness range relevant for affecting cellular interactions covers a wide range from kPa,^[^ ^8a,8b,8n^ ^]^ MPa,^[^ ^8a,8q^ ^]^ to GPa.^[^ ^8g,8n,8q^ ^]^ The differences in the relevant stiffness reported in these studies may arise from the application of different cell models, targeting of different receptors that leads to uptake through different mechanisms, or from differences in the definition of stiffness and in the method used to quantify it. Furthermore, differences in the hydration and temperature of the NPs during the measurements can affect the phase of the polymer (glassy vs rubbery) with important consequences for the stiffness of the NPs. In the case of GM3‐CD169‐mediated NP internalization, the role of the physical properties of the NP core in general and of the stiffness in particular remain insufficiently understood, motivating additional studies under well‐defined conditions.

In this work, we characterize the intracellular fate of GM3‐presenting NPs that have similar size and surface composition but differ in their core stiffness in CD169‐expressing macrophages. We chose this cell model as it i) has high relevance in the context of HIV‐1 transinfection^[^ ^9^ ^]^ and ii) is an important general target for NP delivery. Our experimental strategy to probe the effect of core stiffness is based on lipid‐wrapped polymer NPs, which have also been referred to as core–shell‐type lipid‐polymer hybrid NPs.^[^ ^10^ ^]^ These NPs allow for an uncomplicated presentation of the ganglioside GM3 and facilitate a variation of the core stiffness through choice of core composition. The lipid‐wrapped NPs used in this work have an average diameter of 146 ± 17 and polymer core Young's moduli between 60 ± 32 and 86 ± 25 MPa, which is sufficiently soft to allow different degrees of deformation in response to typical cellular forces in the nN range,^[^ ^11^ ^]^ and 1.41 ± 0.67 GPa as “stiff” NP controls. We investigate the effect of the core stiffness on binding, uptake, and the intracellular distribution of GM3‐functionalized polymer NPs and validate the hypothesis that the unique GM3‐CD169 binding induced spatial segregation patterns observed for both HIV‐1 and HIV‐1‐mimicking NPs requires not only the recognition of the ligand through the receptor but that it also depends on the stiffness of the NP core.

## 2 Results and Discussion

### 2.1 Fabrication and Characterization of Lipid‐Wrapped Polymer NPs

**Figure 1:**
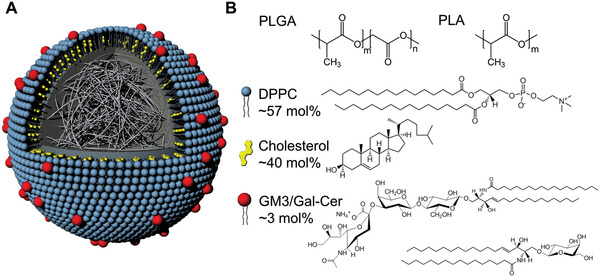
Structure and composition of lipid‐wrapped polymer NPs. A) Schematic drawing of the NP structure. Hydrophobic interactions between NP polymer core and hydrocarbon chains of the lipids result in a lipid monolayer (“membrane”) around the NP core. B) Chemical structure of the polymers in the NP core (PLGA and PLA) and of membrane components (DPPC, cholesterol, GM3, or Gal‐Cer). Gal‐Cer has a similar structure as GM3 but is lacking the sialic acid and, therefore does not bind to the sialic acid recognizing CD169. For lipids the mol% used for the membrane assembly is specified.

Lipid‐wrapped polymer NPs were synthesized in a single step through nanoprecipitation of polymers in the presence of a lipid mix of defined composition through sonication. The assembly of a lipid monolayer, referred to in the following as membrane, around the polymeric NP core is driven by hydrophobic interactions between the lipid tails and the polymer core.^[^ ^12^ ^]^ As core materials, we used ester‐terminated poly(lactic‐*co*‐glycolic) acid (PLGA) with an intrinsic viscosity of 0.32–0.44 dL g^−1^ and a molecular weight (MW) of 24 000–38 000 g mol^−1^ and polylactic acid (PLA) with an intrinsic viscosity of either 0.25–0.35 dL g^−1^ and a MW of 18 000–28 000 g mol^−1^ (low molecular weight PLA, PLA^lMW^) or 1.3–1.7 dL g^−1^ and a MW of 209 000 (high molecular weight PLA, PLA^hMW^). Unless otherwise noted, the membrane was assembled from 56 to 59 mol% 1,2‐dipalmitoyl‐*sn*‐glycero‐3‐phosphocholine (DPPC), 40 mol% cholesterol, either 3 mol% ganglioside GM3, or *α*‐Galactosylceramide (Gal‐Cer), and small amounts of fluorescently labeled phosphatidylethanolamine (PE) lipid, 1,2‐dipalmitoyl‐*sn*‐glycero‐3‐phosphoethanolamine‐*N*‐(lissamine rhodamine B sulfonyl) (ammonium salt) (Liss Rhod PE). This lipid composition was chosen as a minimalistic model to capture all the main properties of the HIV‐1 membrane.^[^ ^4a^ ^]^ Gal‐Cer, which has a similar structure as GM3 but lacks sialic acids and, therefore, does not bind to CD169 was used as a control to test that the NP binding is GM3‐specific. The fluorescent lipid was added to allow detection of the NPs through fluorescence. The structure and composition of a lipid‐wrapped polymer NP is schematically depicted in **Figure** **1**.

**Figure 2:**
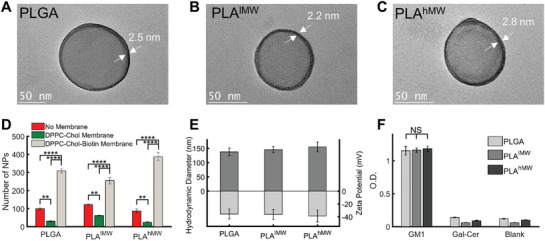
Characterization of lipid‐wrapped polymer NPs. A–C) High‐resolution TEM images of GM3‐functionalized PLGA, PLA^lMW^, and PLA^hMW^ NPs. Samples were treated with sodium phosphotungstate (1% w/v in water). White arrows indicate the lipid membrane around the polymer cores. D) Number of NPs bound to a BSA‐Biotin NeutrAvidin treated surface for NPs with and without biotin containing lipids in the membrane as well as for the no membrane controls. For each condition the average number of bound NPs was determined from 5 separate field‐of‐views, error bars represent standard error of the mean (SEM). E) Hydrodynamic diameter and zeta potential of GM3‐presenting PLGA, PLA^lMW^, and PLA^hMW^ NPs. Error bars represent standard deviation. F) Relative ganglioside (GM1) concentration on the investigated NPs quantified by ELISA performed in triplicates (*n* = 3). Gal‐Cer‐presenting or blank NPs were included as controls. Error bars represent standard deviation. (Statistical *p*‐values are determined using one‐way ANOVA followed by a Tukey post‐hoc test, **p ≤ 0.01, ****p ≤ 0.0001, NS, not significant).

High‐resolution transmission electron microscopy (TEM) images of GM3‐functionalized PLGA, PLA^lMW^, and PLA^hMW^ NPs treated with sodium phosphotungstate Na_3_P(W_3_O_10_)_4_ are shown in **Figure** **2**A–C. Additional TEM images of larger fields of view of sodium phosphotungstate treated lipid‐wrapped polymer NPs and energy dispersive X‐ray spectra are included in Figures S1 and S2 (Supporting Information). Consistent with a successful wrapping of the NPs,^[^ ^8^, ^10^, ^13^ ^]^ we observed an accumulation of stain at the periphery of lipid‐wrapped polymer NPs (marked with arrows in Figure 2A–C) which is not detected for the polymer core NPs without membrane (Figure S3, Supporting Information). The homogenous contrast along the NP periphery in the high‐resolution TEM images suggests that the encapsulation of the NP core in lipids is complete. We also characterized the lipid‐wrapped NPs through optical colocalization of the signals from fluorescently labeled membrane and polymer core (Figure S4, Supporting Information). Optical colocalization of polymer core and membrane confirms a successful wrapping of the polymer core.

To further validate the successful assembly of a lipid membrane around the polymer (PLGA, PLA^lMW^, and PLA^hMW^) NP cores, we included 3 mol% biotin‐functionalized lipids (1,2‐distearoyl‐*sn*‐glycero‐3‐phosphoethanolamine‐*N*‐[biotinyl(polyethyleneglycol)‐2000] (ammonium salt) (DSPE‐PEG‐Biotin)) in the membrane mix and tested the binding for NPs without membrane as well as for NPs wrapped in a DPPC, cholesterol membrane with and without biotin (Figure 2D). We observed low nonspecific binding of the polymer NPs that decreased after assembly of the passivating membrane but then increased strongly in the presence of biotin‐containing lipids. Overall, the observed binding behavior is consistent with a successful formation of a lipid membrane around the polymer cores.

The hydrodynamic diameters, as determined by dynamic light scattering (DLS), for the lipid‐wrapped polymer NPs are 138 ± 13 nm (PLGA), 145 ± 11 nm (PLA^lMW^), and 155 ± 17 nm (PLA^hMW^), with an average polydispersity index < 0.28. The average diameter for all lipid‐wrapped NPs of 146 ± 17 nm is in excellent agreement with the typical diameter of HIV‐1 particles.^[^ ^14^ ^]^ The zeta potentials of the GM3‐functionalized PLGA (−35 ± 8 mV), PLA^lMW^ (−36 ± 8 mV), and PLA^hMW^ (−39 ± 9 mV) NPs confirmed statistically indistinguishable surface charges. The hydrodynamic diameters and zeta potentials of GM3‐presenting PLGA, PLA^lMW^, and PLA^hMW^ are summarized in Figure 2E. Polymer NP cores that were formed through nanoprecipitation in the absence of lipids had average hydrodynamic diameters of 80 ± 7 nm (PLGA), 120 ± 16 nm (PLA^lMW^), 123 ± 8 (PLA^hMW^), and zeta potentials of −27 ± 9 mV (PLGA), −15 ± 5 mV (PLA^lMW^), and −24 ± 8 mV (PLA^hMW^).

The concentration of the ganglioside GM3 in the membrane around the NP core plays an important role in targeting CD169 and the subsequent cellular response induced by NP binding.^[^ ^15^ ^]^ To avoid systematic biases in NP uptake and trafficking due to differences in the ganglioside content of the membrane around the different polymer cores, it is important to ensure equal ganglioside loadings for all polymer NPs. We characterized the loading of the structurally closely related ganglioside GM1 by ELISA (Figure 2F). We detected virtually identical GM1 levels for PLGA, PLA^lMW^, and PLA^hMW^ NP cores, confirming that the nature of the NP core does not affect the ganglioside concentration loaded onto the NPs.

In summary, our characterization data reveal that PLGA, PLA^lMW^, and PLA^hMW^ NPs have nearly identical sizes of ≈146 ± 17 nm and are successfully wrapped in lipid membranes with identical ganglioside concentration and surface charge.

The lipid‐wrapped NPs are stable under typical cell culture conditions in vitro. We confirmed that GM3‐functionalized NPs do not agglomerate in 10% fetal bovine serum (FBS) RPMI for at least 24 h (Figure S5, Supporting Information). To validate the stability of the NPs under simulated intracellular conditions, we incubated surface‐immobilized fluorescently labeled lipid‐wrapped NPs with cell lysate and imaged the same field of view immediately after addition of the cell lysate as well as after different incubation times (Figure S6, Supporting Information). The lipid‐wrapped NPs were stable for the duration of the experiment of 5 days.

Finally, we evaluated the cytotoxicity of the lipid‐wrapped NPs under the experimental conditions of this study with a 3‐(4,5‐dimethylthiazol‐2‐yl)‐2,5‐diphenylterazolium bromide (MTT) assay (Figure S7, Supporting Information). No cytotoxicity was detected.

### 2.2 Characterizing Glass Transition Temperature and Stiffness of Polymer NPs

**Figure 3:**
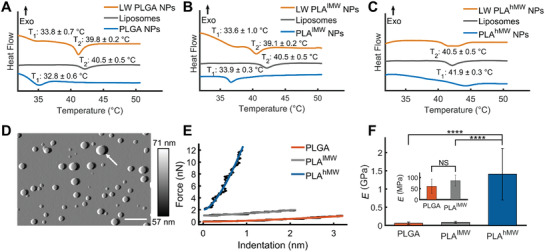
Thermal analysis and stiffness characterization of lipid‐wrapped polymer NPs. A–C) DSC thermograms of PLGA, PLA^lMW^, and PLA^hMW^ NPs. In each plot there are three thermograms which correspond to polymer NPs (without membrane), liposomes, and lipid‐wrapped (LW) polymer NPs. T_1_ and T_2_ represent glass transition and phase transition temperature of polymer core and lipid membrane, respectively. DSC software was used to determine the glass transition and phase transition temperatures, the onset values are reported on the plots. All DSC experiments were performed at least as triplicates. D) Representative atomic force microscopy (AFM) tapping mode amplitude map of dispersed PLA^lMW^ NPs in water at 37 °C, scale bar = 500 nm, and amplitude colorbar range from 57 to 71 nm. The tallest NP height in the scan (as denoted by white arrow) is measured from the height channel from the same scan as 174.6 nm. E) Force‐indentation curves of representative PLGA, PLA^lMW^, and PLA^hMW^ NPs with overlaying modified Hertzian fits. AFM force measurements were performed on hydrated NPs in water at 37 °C. PLA^lMW^ and PLA^hMW^ force curves are offset vertically by 1 and 2 nN, respectively, for visual clarity. F) The average measured Young's modulus (*E*) of PLGA, PLA^lMW^, and PLA^hMW^ NPs, values represent means of at least *n* = 11 NPs. Error bar represents standard deviation. (Statistical *p*‐values are determined using one‐way ANOVA followed by a Tukey post‐hoc test, ****p ≤ 0.0001, NS, not significant).

Differential scanning calorimetry (DSC) was used to detect the glass and phase transition temperatures in NPs, membrane, and lipid‐wrapped NPs. In these experiments we adjusted the composition of the lipid membrane to 10 mol% cholesterol and 90 mol% DPPC to allow for a detectable phase transition from solid ordered to liquid disordered membrane.^[^ ^16^ ^]^ The thermograms of polymer NPs without any membrane and liposomes of identical lipid composition each have one endothermic peak (**Figure** **3**A–C), which corresponds to the glass transition temperature of the polymer and the phase transition temperature of the lipid membrane in liposomes, respectively. The glass transition temperatures for PLGA, PLA^lMW^, and PLA^hMW^ were determined as 32.8 ± 0.6, 33.9 ± 0.3, and 41.9 ± 0.3 °C. The phase transition temperature of liposomes was found to be 40.5 ± 0.5 °C. Importantly, the thermograms of lipid‐wrapped polymer NPs show two peaks for PLGA and PLA^lMW^ cores (Figure 3A,B). Consistent with the lipid‐wrapped polymer NP design, one peak aligns well with the glass transition temperature of the polymer core, while the second peak at higher temperature is close to the phase transition temperature of the lipid membrane. For PLA^hMW^ NPs, glass transition temperature and phase transition temperature occur at nearly the same temperature and cannot be resolved. We attribute the broad feature in the PLA^hMW^ thermogram to the coexistence of the two transitions at around 40–44 °C (Figure 3C). The observations of membrane‐ and core‐associated phase transitions in Figure 3A–C provide further experimental evidence of a successful membrane assembly around the polymer NP cores.

Thermograms of dried polymer NPs are provided in Figure S8A (Supporting Information), the glass transition temperatures of dried polymer NPs are in the range of 39–49 °C. We also measured the glass transition temperatures of bulk PLGA, PLA^lMW^, and PLA^hMW^ (as powders) by DSC (Figure S8B, Supporting Information), these values were in the range of 43–51 °C. The glass transition temperatures for dried polymer NPs as well as for bulk polymers are appreciably higher than those of their NPs in solution. This discrepancy is explained by the plasticizing effect of water in the polymer NPs. Even a relatively small amount of water, 3–5 wt%, in polymer NPs can induce a significant decrease in the glass transition temperature. The depression of the glass transition temperature in water containing polymer NPs is well described by the Gordon–Taylor relationship.^[^ ^17^ ^]^

The lipid‐wrapped NPs investigated in this work can be subdivided into two groups: NPs with a polymer core whose glass transition temperature in solution lies below 37 °C (PLGA and PLA^lMW^) (Figure 3A,B) or above 37 °C (PLA^hMW^) (Figure 3C). Consequently, at the physiological temperatures of the cell experiments, only PLA^hMW^ NPs remain in the glassy state, while all other polymers are in the rubbery state. The intrinsic elasticity, described by the Young's modulus (*E*), determines the stiffness of a NP with a given size and shape. We measured the Young's moduli of individual PLGA, PLA^lMW^, and PLA^hMW^ NPs immobilized on a glass substrate in water at 37 °C using atomic force microscopy (AFM). A typical AFM image of PLA^lMW^ NPs is depicted in Figure 3D. AFM images of PLGA and PLA^hMW^ NPs as well as schemes of the experimental approach used for scanning and nanoindentation measurements are provided in Figure S9 (Supporting Information). Representative force versus indentation curves for PLGA, PLA^lMW^, and PLA^hMW^ NPs are shown in Figure 3E. Nanoindentation curves obtained from individual NPs were fit using a modified Hertzian model that incorporates the comparable size of the spherical indenter to that of the nanoparticle and the planar substrate (see the Experimental section). The average Young's moduli obtained from the fits (at least 11 NPs per conditions) are: *E*_PLGA_ = 60 ± 32 MPa, *E*_PLA_^lMW^ = 86 ± 25 MPa, and *E*_PLA_^hMW^ = 1.41 ± 0.67 GPa (Figure 3F). The differences between the Young's moduli of PLGA/PLA^lMW^ and PLA^hMW^are significant at a *p*‐value < 0.0001.

The observed difference in the stiffness of the investigated polymer cores (PLGA, PLA^lMW^, and PLA^hMW^) with different phase states at physiological conditions makes it possible to probe the fate of NPs with a hard or rubbery core upon GM3‐CD169‐mediated uptake with lipid‐wrapped NPs.

### 2.3 GM3‐CD169‐Mediated Binding of Lipid‐Wrapped PLGA, PLA^lMW^, and PLA^hMW^ NPs to THP‐1 CD169^+^ Cells

CD169^+^ THP‐1 cells (monocytes) were incubated with lipid‐wrapped NPs containing GM3 or Gal‐Cer, or without glycosphingolipids (blank) controls for 60 min at a NP : cell ratio of 1 × 10^6^ : 1.

**Figure 4:**
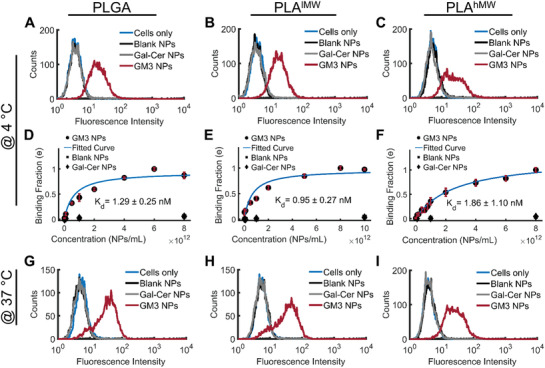
Specificity of GM3‐CD169 binding of polymer NPs and binding curves obtained with undifferentiated CD169^+^ THP‐1 cells. A–C) Histograms of fluorescence intensity of NP binding after 60 min incubation at 4 °C. Conditions include GM3‐ or Gal‐Cer‐presenting NPs, or blank NPs with A) PLGA, B) PLA^lMW^, and C) PLA^hMW^ cores. D,F) Relative binding (normalized geometric‐mean of fluorescence intensity histograms) of CD169^+^ THP‐1 cells after 60 min incubation at 4 °C. Conditions include GM3‐ or Gal‐Cer‐presenting NPs, or blank NPs with D) PLGA, E) PLA^lMW^, and F) PLA^hMW^ cores. G–I) Histograms of fluorescence intensity of NP binding after 10 min incubation at 37 °C. Conditions include GM3‐ or Gal‐Cer‐presenting NPs, or blank NPs with G) PLGA, H) PLA^lMW^, and I) PLA^hMW^ cores. All experiments were performed as triplicates and error bars represent SEM.

In a first set of experiments we incubated NPs with cells at 4 °C to minimize active NP uptake. The cells were washed after NP exposure and fixed in 4% paraformaldehyde (PFA) before NP binding was measured via flow cytometry. Independent of the choice of the polymer core, only GM3‐presenting NPs but not Gal‐Cer‐presenting NPs or blank controls bound to the CD169‐expressing cells, confirming that the binding of all investigated NPs is GM3‐specific (**Figure** **4**A–C).

Next, we varied the NP input concentration between 10^8^ and 10^13^ NPs mL^−1^ (corresponding to NP:cell ratios between 1000:1–10^8^:1) and recorded binding curves for the lipid‐wrapped NP binding to CD169^+^ THP‐1 cells (Figure 4D–F). While Gal‐Cer and blank controls show negligible binding at all concentrations, GM3‐presenting NPs show a concentration‐dependent increase in binding to CD169^+^ THP‐1 cells. The binding curves for GM3‐presenting PLGA, PLA^lMW^, and PLA^hMW^ NPs are well described by Langmuir isotherm fits (included as continuous lines) with dissociation constants, *K*_d_, (average of three independent binding experiments) of *K*_d_ = 1.29 ± 0.25 × 10^−9^ m for PLGA, 0.95 ± 0.27 × 10^−9^ m for PLA^lMW^, and 1.86 ± 1.10 × 10^−9^ m for PLA^hMW^. With a dissociation constant for individual GM3‐CD169 contacts in the 1 × 10^−3^ m range,^[^ ^9^, ^18^ ^]^ the observed *K*_d_ values suggest a strong multivalent amplification of the binding avidity.^[^ ^15^ ^]^ The differences between *K*_d_ values of PLGA, PLA^lMW^, and PLA^hMW^ NPs are statistically not significant, indicating that the binding affinity of the GM3‐presenting NPs is determined by GM3‐CD169 recognition and does not significantly depend on the choice of the polymer as core material. Since at 4 °C all investigated polymers are rigid and in the glassy state, which might limit the role of the NP core in affecting binding affinities, we performed additional binding experiments at 37 °C with a limited incubation time of 10 min (Figure 4G–I). For this short incubation time, the measured NP signal is mostly dominated by binding to cell surface expressed CD169. At 37 °C all NP cores with the exception of PLA^hMW^ are in their rubbery state. We observed again nearly identical binding for all NPs, which further confirms that the initial binding of GM3‐displaying NPs to CD169 is independent of the core stiffness.

### 2.4 Uptake of GM3‐Presenting PLGA, PLA^lMW^, and PLA^hMW^ NPs by CD169^+^ Macrophages

**Figure 5:**
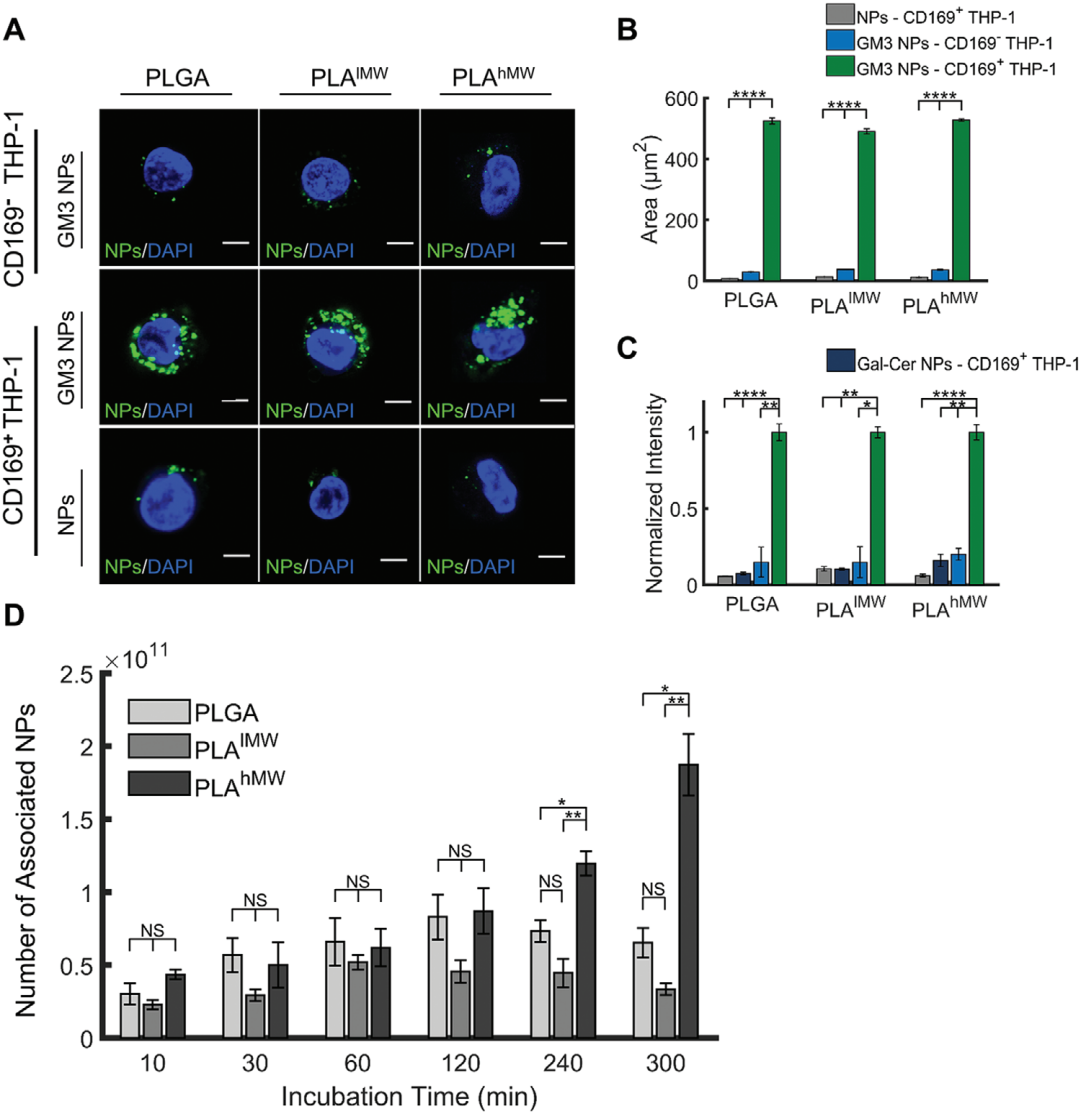
Specific binding and cellular uptake of GM3‐presenting PLGA, PLA^lMW^, and PLA^hMW^ core NPs in CD169‐expressing macrophages (differentiated THP‐1 cells). A) Confocal sections of CD169^+^ and CD169^−^ THP‐1 macrophages incubated with fluorescent PLGA, PLA^lMW^, and PLA^hMW^ NPs (without membrane, no GM3) and GM3‐presenting PLGA, PLA^lMW^, and PLA^hMW^ NPs for 10 min at 37 °C. Scale bar = 5 µm. B) Integrated NP‐containing area in 25 CD169^+^ and CD169^−^ cells. C) Normalized fluorescence intensities of CD169^+^/CD169^−^ THP‐1 cell lysates after 10 min incubation at 37 °C with PLGA, PLA^lMW^, and PLA^hMW^ NPs without GM3 (and membrane), with GM3‐containing membrane, and with Gal‐Cer‐containing membrane. D) Number of associated GM3‐presenting PLGA, PLA^lMW^, and PLA^hMW^ NPs with CD169^+^ macrophages after specified incubation times at 37 °C. NP concentration was 5 × 10^12^ NPs mL^−1^ throughout. The average of three independent experiments is plotted in (B–D), and error bars represent the SEM. (Statistical *p*‐values are determined using one‐way ANOVA followed by a Tukey post‐hoc test, *p ≤ 0.05, **p ≤ 0.01, ****p ≤ 0.0001, NS, not significant).

CD169^+^ THP‐1 cells were differentiated into macrophages using phorbol myristate acetate (PMA).^[^ ^4^, ^19^ ^]^ We first confirmed that the binding of lipid‐wrapped NPs without GM3 to the macrophages was negligible and that GM3‐presenting NPs did not bind to CD169^−^ macrophages (**Figure** **5**A–C). Next, GM3‐presenting PLGA, PLA^lMW^, and PLA^hMW^ NPs were incubated with the cells at the concentration of 5 × 10^12^ NPs mL^−1^ in serum‐containing medium at 37 °C for time durations between 10 and 300 min.

After that, we determined the fluorescence intensity of the cell lysates obtained from the adherent cells using a plate reader. Figure 5D summarizes the association (binding and uptake) of NPs with cells measured at different time points. For incubation times ≤ 120 min, we observe a moderate increase in the number of associated NPs as a function of time, but there is no statistically significant difference between PLGA, PLA^lMW^, or PLA^hMW^ NP cores. However, differences become detectable at longer incubation times. After 240 and 300 min, PLA^hMW^ NPs show a statistically significant higher association than PLGA and PLA^lMW^ NPs. For long incubation times differences in association will be determined by uptake. Consequently, the observed differences between NPs with identical lipid wrap but different polymer cores indicate that the core affects GM3‐CD169‐mediated internalization. A higher uptake for PLA^hMW^ NPs, which is the stiffest among the investigated NPs, is congruent with the majority of previous studies^[^ ^8a,8c,8g,8n,8q^ ^]^ characterizing the role of NP stiffness in uptake and corroborates the hypothesis that core stiffness influences GM3‐CD169 mediated uptake. Only a few studies have reported increased uptake for softer NPs^[^ ^8d,8f^ ^]^ or for NPs with intermediate elastic moduli.^[^ ^8b^ ^]^ Development of a robust difference in the association of GM3‐presenting NPs with CD169^+^ macrophages for different polymer cores is relatively slow (Figure 5D). It takes more than 2 h before the increase for the PLA^hMW^ NPs become statistically significant. The time‐scale of the process, which is in general agreement with that reported by Anselmo et al.^[^ ^8a^ ^]^ and Banquy et al.^[^ ^8b^ ^]^ in their studies of the effect of core stiffness on the uptake of hydrogel NPs, suggests that relatively slow cellular processes are responsible for the observed core‐specific uptake differences.

Several factors can account for a preferential uptake of GM3‐presenting NPs with a stiffer core. One factor that relates to NP‐cell interactions in general is cell‐induced morphological change of NPs during uptake. Computational analyses of the elastic deformation energy as a function of surface wrapping illustrated that in order to accomplish full NP wrapping, which is a key step during uptake, ellipsoidal NPs require up to 30% more energy than spherical NPs.^[^ ^8g^ ^]^ Experimental studies investigating the uptake of nm to µm‐sized particles confirmed that spherical NPs are in general more efficiently uptaken than elongated ones.^[^ ^6^, ^20^ ^]^ Stiff NPs, such as PLA^hMW^ NPs in our studies, show a higher resistance against structural deformations than softer PLGA or PLA^lMW^ NPs, which may enhance uptake. Another effect that couples NP stiffness to uptake efficiency, especially in the case of actin‐dependent phagocytosis, is a failure of soft NPs to induce the formation of actin filaments.^[^ ^8c^ ^]^ This point is of particular interest for HIV‐1‐mimicking NPs as actin also plays an important role at various stages of the HIV‐1 infection.^[^ ^21^ ^]^

Perturbation or modification of actin‐driven internalization and subsequent trafficking of GM3‐presenting NPs with a soft core may result in a gradual accumulation of stiffness‐dependent differences in total intracellular NP content.

### 2.5 Characterization of the Intracellular Fate and Spatiotemporal Distribution of GM3‐Presenting PLGA, PLA^lMW^, and PLA^hMW^ NPs in CD169^+^ Macrophages

To characterize the intracellular fate of GM3‐presenting NPs with different cores, we mapped the intracellular distribution of NPs with PLGA, PLA^lMW^, and PLA^hMW^ cores in CD169‐expressing macrophages (CD169^+^ THP‐1) through confocal fluorescence microscopy. Cells were incubated with NPs for 10 min followed by a chase of 16 h. The rationale for this approach is that potential differences in the uptake mechanism(s) triggered by different NP cores can be distinguished through differentiable spatiotemporal distributions of the NPs. It has previously been shown that GM3‐CD169 mediated binding of HIV‐1 to macrophages is instrumental in avoiding an endolysosomal pathway as it can trigger collection of virus particles in specialized, nonlysosomal, but tetraspanin CD9‐positive compartments that are commonly referred to as virus‐containing compartments (VCCs).^[^ ^22^ ^]^

**Figure 6:**
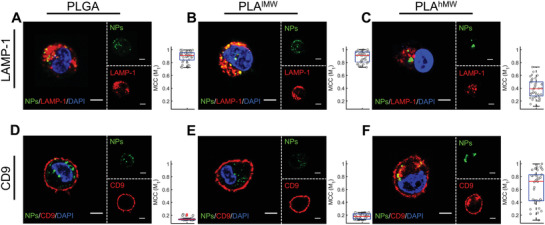
Mapping the intracellular fate of GM3‐presenting PLGA, PLA^lMW^, and PLA^hMW^ NPs in CD169‐expressing macrophages after 10 min of incubation with NPs and subsequent chase of 16 h and staining for LAMP‐1 and CD9. A–C) Confocal sections of macrophages stained for LAMP‐1. LAMP‐1 was fluorescently labeled as lysosome marker. D–F) Confocal sections of macrophages NPs with immunolabeled CD9. CD9 was fluorescently labeled as VCC marker. M_1_ values represent Manders’ Colocalization Coefficients for the overlap of NP signal with the marker of interest. The M_1_ values of 40 randomly selected cells are included as the box plots. Scale bar = 5 µm.

We tested for colocalization of GM3‐presenting NPs with lysosomes and VCCs using lysosomal‐associated membrane protein 1 (LAMP‐1) and CD9 as markers.^[^ ^23^ ^]^ Confocal sections of the dominant cell phenotypes obtained after staining with LAMP‐1 and CD9 are shown in **Figure** **6** (additional examples are shown in Figures S10 and S11, Supporting Information). For GM3‐presenting PLGA and PLA^lMW^ NPs we observed a predominant localization in lysosomes (Figure 6A,B). Intriguingly, for GM3‐presenting PLA^hMW^ NPs we found that a significant fraction of NPs did not colocalize with the lysosome marker (Figure 6C). Differences between the soft PLGA, PLA^lMW^ NPs, and the stiffer PLA^hMW^ NPs were also prevalent in the CD9 staining patterns. For cells incubated with PLGA or PLA^lMW^, CD9 staining was confined to the cell periphery, where the NP concentration was low after 16 h. Consequently, the colocalization of PLGA and PLA^lMW^ NPs with CD9 was very low (Figure 6D,E). In contrast, in cells incubated with PLA^hMW^ NPs we frequently observed NP localization within intracellular CD9‐positive compartments (Figure 6F).

Next, we quantified the colocalization of polymer NPs with LAMP‐1 and CD9 by calculating Manders’ colocalization coefficients (MCC).^[^ ^24^ ^]^ M_1_ quantifies the overlap of NP and respective marker (LAMP‐1 or CD9) signal, and M_2_ characterizes the overlap of the marker signal with the NP signal. For LAMP‐1 the average MCC values in 40 randomly selected cells per conditions are M_1_ = 0.89 ± 0.08, M_2_ = 0.15 ± 0.15 (PLGA); M_1_ = 0.88 ± 0.09, M_2_ = 0.23 ± 0.15 (PLA^lMW^); M_1_ = 0.41 ± 0.15, M_2_ = 0.20 ± 0.18 (PLA^hMW^) and for CD9 we found average MCC values of M_1_ = 0.02 ± 0.02, M_2_ = 0.01 ± 0.01 (PLGA); M_1_ = 0.06 ± 0.04, M_2_ = 0.01 ± 0.01 (PLA^lMW^); M_1_ = 0.60 ± 0.28, M_2_ = 0.06 ± 0.03 (PLA^hMW^). Box plots of the M_1_ values for 40 randomly selected cells per staining conditions are included in Figure 6. PLGA and PLA^lMW^ NPs show good colocalization with LAMP‐1 and only negligible overlap with CD9, but for PLA^hMW^ the colocalization between NPs and CD9 is increased, whereas the colocalization between NPs and LAMP‐1 is decreased. The average M_1_ value for the NP colocalization with LAMP‐1 is significantly lower (*p*‐value < 0.0001) for PLA^hMW^ when compared with PLGA and PLA^lMW^ but significantly higher (*p*‐value < 0.0001) in the case of CD9. Intriguingly, the distribution of the M_1_ values for PLA^hMW^ NPs and CD9 (median = 0.70) as shown in the box plot in Figure 6F exhibits a bimodal distribution with two groups of M_1_ values. The first one clustered at around M_1_ = 0.36 indicates cells with moderate NP‐CD9 colocalization, but the second group of cells clustered at around M_1_ = 0.84 shows a strong optical colocalization of the signals from PLA^hMW^ NPs and CD9. A localization of PLA^hMW^ NPs in CD9‐positive, nonlysosomal compartments in a significant fraction of the cell population accounts naturally for the drop in colocalization with LAMP‐1. The M_1_ values for the colocalization of PLA^hMW^ NPs with LAMP‐1 lie below 0.41 for approximately half of the cell population (see box plot in Figure 6C).

Overall, the systematic differences in the M_1_ values for GM3‐presenting NPs with PLA^hMW^ or PLGA, PLA^lMW^ cores corroborate different intracellular fates for the two groups. PLGA, PLA^lMW^ NPs are exclusively collected in lysosomes, but PLA^hMW^ are trafficked via a different pathway into CD9‐positive compartments in a significant fraction of the cells. The staining pattern of the GM3‐presenting PLA^hMW^ NPs in Figure 6; and Figures S10 and S11 (Supporting Information) with a localization in CD9‐positive and LAMP‐1‐negative compartments is reminiscent of the collection of virus particles in VCCs in macrophages. We also validated that this compartmentalization was not unique to the THP‐1 cell lines by repeating the experiment with human monocyte derived macrophages (MDMs). Figure S12 (Supporting Information) shows confocal sections of MDMs stained for CD169 (Figure S12A, Supporting Information), CD9 (Figure S12B, Supporting Information), and LAMP‐1 (Figure S12C, Supporting Information). In good agreement with findings in CD169^+^ THP‐1 cells, we observed a collection of GM3‐presenting PLA^hMW^ NPs in compartments that optically colocalize with CD9 and CD169 but not the lysosome marker LAMP‐1.

The lower number of intracellular NPs in Figure 6; and Figure S10–S12 (Supporting Information) compared to those of that in Figure 5A is attributed to the longer chasing time of 16 h in Figure 6; and Figures S10–S12 (Supporting Information) versus no chasing time in Figure 5A. Intracellular degradation as well as trafficking during the chase time can account for a lower number of discrete NPs. In the case of GM3‐presenting PLA^hMW^ NPs with the stiffest core, sequestration of NPs in VCCs can explain the seemingly lower number of intracellular NPs. We have shown previously in a study with stiff gold GM3‐NPs in dendritic cells that initially randomly distributed GM3‐NPs are eventually collected in a central VCC‐like compartment, resulting in a lower number of optically discernable “particles”per cell.^[^ ^4b^ ^]^

The increase in compartmentalization in nonlysosomal compartments observed for PLA^hMW^ NPs in Figure 6; and Figure S12 (Supporting Information) suggests that this intracellular fate is favored by higher NP core stiffness. To address the question whether high core stiffness is sufficient to trigger selective compartmentalization of NPs in nonlysosomal compartments, independent of GM3‐CD169 binding, we mapped the intracellular fate of lipid‐wrapped PLGA, PLA^lMW^, and PLA^hMW^ NPs with a modified membrane that did not contain GM3 but phosphatidylserine (PS) (50 mol% DPPC, 40 mol% cholesterol, 10 mol% 1,2‐dioleoyl‐*sn*‐glycero‐3‐phospho‐*L*‐serine (sodium salt) (DOPS)). The negatively charged PS allows NPs to bind to scavenger receptors^[^ ^25^ ^]^ as well as specialized PS receptors, such as Tyro3, Axl, and Mer (TAM)^[^ ^26^ ^]^ or T cell/transmembrane, immunoglobulin, and mucin (TIM)^[^ ^27^ ^]^ family receptors. Importantly, all of the GM3‐free NPs, regardless of the nature of the polymer core, were found to accumulate in the lysosomal compartments (Figure S13, Supporting Information). We conclude that the NP core properties by themselves are insufficient to induce the sequestration of NPs in nonlysosomal but CD9‐positive compartments.

The fact that both GM3 ligand and PLA^hMW^ cores are required to induce sequestration in nonlysosomal compartments indicates that biochemical recognition of GM3 through CD169 and mechanical recognition of the NP core interact synergistically to trigger cargo sequestration in nonlysosomal compartments.

## 3 Conclusion

In this work, we have investigated the intracellular fates of ganglioside GM3‐presenting lipid‐wrapped polymer NPs with different polymer cores in CD169‐expressing macrophages. Core materials included PLGA (MW 24 000–38 000 g mol^−1^) and PLA with two molecular weights (MW 18 000–28 000 g mol^−1^ (PLA^lMW^) and 209 000 g mol^−1^ (PLA^hMW^). The lipid‐wrapped NPs resembled each other in shape, ganglioside concentration, and surface composition, and had similar hydrodynamic diameters but differed in the glass transition temperature and stiffness of the NP core. Only PLA^hMW^ NPs had a glass transition temperature above 37 °C in solution. Due to the higher glass transition temperature, PLA^hMW^ NPs were much stiffer under physiological conditions than PLGA or PLA^lMW^ NPs. Nanoindentation experiments revealed a Young's modulus of *E*_PLA_^hMW^ = 1.41 ± 0.67 GPa, compared to *E*_PLA_^lMW^ = 86 ± 25 MPa and *E*_PLGA_ = 60 ± 32 MPa, for hydrated NPs at 37 °C.

The differences in the core stiffnesses were associated with different uptake behaviors and intracellular fates. GM3‐presenting NPs with PLA^hMW^ cores showed a higher cellular uptake than GM3‐presenting NPs with PLA^lMW^ or PLGA cores under condition of continuous incubation. A characterization of the spatial distribution of GM3‐presenting PLGA, PLA^lMW^, and PLA^hMW^ NPs in THP‐1 cells revealed that GM3‐presenting NPs with PLA^hMW^ cores but not the softer PLA^lMW^ or PLGA cores, were sequestered in nonlysosomal compartments. While PLA^hMW^ NPs were preferentially collected in nonlysosomal, CD9‐positive compartments, GM3‐presenting PLGA and PLA^lMW^ NPs were almost exclusively collected in lysosomes. The nonlysosomal PLA^hMW^ NPs containing compartments shared distinct similarities with VCCs in CD169‐expressing macrophages,^[^ ^4^, ^22^ ^]^ including the enrichment of tetraspanin CD9. We validated in control experiments with PS‐containing PLA^hMW^ NPs without GM3 that the spatial sequestration in CD9‐positive compartments required GM3‐CD169 interactions. Considering the fact that all NPs were wrapped in the same membrane, but differed in the stiffness of the polymer cores, our experimental observations suggest that the biochemical recognition of GM3‐presenting NPs is augmented or modified by a mechanical recognition. Only for NPs that present GM3 and with enhanced core stiffness, an alternative uptake and trafficking pathway becomes available that results in sequestration of GM3‐presenting NPs in nonlysosomal CD9 positive compartments in macrophages. These findings are also consistent with different intracellular fates of GM3‐presenting gold NPs (*E* ≈ 60 GPa^[^ ^28^ ^]^) and liposomes (for which Young's moduli between *E* ≈ 3 kPa^[^ ^29^ ^]^ and 13 MPa^[^ ^30^ ^]^ have been reported) in macrophages.^[^ ^4d^ ^]^

The observed interplay between chemical and mechanical aspects of recognition in the determination of the intracellular fate of the lipid‐wrapped virus‐mimicking NPs is particularly interesting considering an earlier observation of a “stiffness switch” in HIV particles at different stages of the virus life cycle.^[^ ^31^ ^]^ In the previous study, the Young's modulus of mature and immature HIV particles were determined to be 440 and 930 MPa, respectively. Among the NPs investigated in this work, only PLA^hMW^ falls in the “stiff region” of the virus life cycle, which gives additional biological context for the preferential collection of these NPs in VCC‐like compartments. Our findings highlight NP stiffness as a potential regulatory element of NP‐cell interactions and intracellular fates that should be considered in addition to size and multivalency‐related control mechanisms^[^ ^32^ ^]^ in the design of next‐generation artificial NP platforms for applications in nanomedicine.

This work focused on macrophages as cell model. Our findings warrant future studies to test if the same interplay of mechanical and chemical recognition applies to other cell types and to test whether GM3‐presenting NPs can act as delivery platform for the selective delivery of payload to nonlysosomal compartments in antigen presenting cells in vivo.

## 4 Experimental Section

##### Polymer and Lipid‐Wrapped Polymer NP Preparation

Polymer and lipid‐wrapped polymer NPs were prepared through one‐step nanoprecipitation synthesis. A lipid mixture (0.15 mg) containing DPPC (25 mg mL^−1^) and cholesterol (25 mg mL^−1^) and the fluorescence marker 1,2‐dipalmitoyl‐*sn*‐glycero‐3‐phosphoethanolamine‐*N*‐(lissamine rhodamine B sulfonyl) (ammonium salt) (Liss Rhod PE) (1 mg mL^−1^) in chloroform was added to 4 mL of Milli‐Q water. Next, 0.4 mL of PLGA (poly (D,L‐lactide‐*co*‐glycolide) lactide:glycolide 50:50, ester terminated, RG503, with intrinsic viscosity of 0.32–0.44 dL g^−1^ and MW of 24 000–38 000), PLA (poly(D,L‐lactide), ester terminated, R203S with intrinsic viscosity of 0.25–0.35 dL g^−1^ and molecular weight of 18 000–28 000) or high molecular weight PLA (poly(D,L‐lactide) ester terminated, R207S with intrinsic viscosity of 1.3–1.7 dL g^−1^ and molecular weight of 209 000^[^ ^33^ ^]^) solution in acetonitrile was pipetted dropwise to the aqueous solution. Concentration of all the polymer solution is 2.5 mg mL^−1^. The volume ratio of aqueous to organic solution was chosen to be 10:1,^[^ ^12^ ^]^ using a lipid/polymer weight ratio of 15%.^[^ ^10a^ ^]^ To ensure the formation of the lipid membrane around the polymer core, the final solution was sonicated in a bath sonicator (Branson Ultrasonics 5510, Danbury, CT) for 5 min. Next, NPs were washed 3 times (4000g—15 min) using an Amicon Ultra‐4 centrifugal filter (MilliporeSigma, Burlington, MA) with a molecular weight cutoff of 10 kDa to remove organic solvent and free lipid molecules.

To fabricate GM1, GM3, Gal‐Cer, and biotinylated lipid‐wrapped NPs; GM1 ganglioside(Ovine Brain) (2 mg mL^−1^), GM3 ganglioside (Milk, Bovine‐Ammonium Salt) (2 mg mL^−1^), D‐galactosyl‐ß‐1,1′ N‐palmitoyl‐*D*‐erythro‐sphingosine(Gal‐Cer) (2 mg mL^−1^), and 1,2‐distearoyl‐*sn*‐glycero‐3‐phosphoethanolamine‐*N*‐[biotinyl(polyethylene glycol)‐2000] (ammonium salt) (DSPE‐PEG‐Biotin) (10 mg mL^−1^) were added to the lipid mixture, respectively. To generate negatively charged lipid‐wrapped NPs, DOPS (10 mg mL^−1^) was added to the lipid mixture. To incorporate the hydrophobic dye into the core of NPs, 7‐Methoxycoumarin‐3‐carboxylic acid, succinimidyl ester (ATT Bioquest, Inc., Sunnyvale, CA) was added to the polymer solution before the nanoprecipitation process. Polymer NPs without membrane were obtained following the same procedure in the absence of lipids. DLS was applied to monitor the quality of the samples.

All lipids were purchased from Avanti Polar Lipids (Alabaster, AL). Chloroform, acetonitrile, and all the polymers (PLGA‐PLA^lMW^‐PLA^hMW^) were purchased from Sigma‐Aldrich (St Louis, MO).

##### AFM Measurements—Experimental Procedure

Samples were prepared by 20 min incubation of polymer NPs (without membrane) on 0.1% poly‐*L*‐lysine (Sigma‐Aldrich, St Louis, MO) treated circular cover slips (Asylum Research). Following the incubation, unbound NPs were removed by successive washes, and inspected via AFM without drying. Surfaces were first placed inside a Peltier heater attachment (Asylum Research, CoolerHeater) integrated with the AFM (Oxford Instruments— MFP3D Infinity AFM) with 2 mL of Milli‐Q water and left to equilibrate with the AFM head in place for 20 min. After this time, the AFM probe was calibrated using quintuplicate indentations on the rigid cover slip (to find the optical lever sensitivity) and a measurement of the thermal power spectral density (to find the spring constant). For measurements on soft PLGA and PLA^lMW^ NPs, probes with stiffness *k* = 0.08 N m^−1^ (Asylum Research, BL‐AC40TS) were used, whereas for the more rigid PLA^hMW^ NPs, probes with stiffness *k* = 0.9 N m^−1^ (MikroMasch, CSC37/Al BS) were used.  In order to visualize the NPs, an area of 3 × 3 µm^2^ was scanned at 1 Hz in tapping mode to locate a sizeable number of particles (Figure 3D; and Figure S9, Supporting Information). Next, the tallest NP in the scan was identified using AFM software, and the diameter of the AFM tip was calculated by subtracting the height of the NP from its measured width. Calculated AFM tip radii ranged from 20 to 40 nm based on duration of use. Once the survey scan had been completed, a magnified area of 300 × 300 nm^2^ was scanned at 3 Hz in tapping mode to image a single NP. The scan rate was increased to prevent interactions between tip and NP from manipulating the NP. The highest point of the NP was once again found using AFM software, and subsequent force curves with a force set point of 1 nN (PLGA and PLA^lMW^) or 10 nN (PLA^hMW^) at 0.04 Hz were carried out at the point of maximum height of the NP. The NP was scanned after indentation to check for movement or drift. If the particle was observed to have moved appreciably as a result of drift during the force testing, the indentation data for that particular NP were not used. The probe was then moved to another location on the surface and the imaging and indentation was repeated. If the tip radius was calculated to be larger than 50 nm, the probe was replaced.

##### Data Processing

Data in the form of force and indentation was exported from the AFM software. The force and indentation were calculated using the calibration of the optical lever sensitivity and stiffness conducted on every probe prior to use.^[^ ^34^ ^]^ To isolate^[^ ^35^ ^]^ the modulus of the NP, the following equation^[^ ^36^ ^]^ was used to calculate the NP modulus

(1) $$F=E_{\mathrm{NP}}\times \left[\frac{4{\left(d\;*\;b\right)}^{\frac{3}{2}}R_{t,\mathrm{NP}}^{\frac{1}{2}}}{3\left(1-\nu_{\mathrm{NP}}^{2}\right)}\right]$$

where *F* is the force exerted by the AFM probe, *E*_NP_ is the modulus of the NP, *d* is the indentation depth measured by the AFM, and *ν* is the Poisson's ratio of the NP. Poisson's ratio for PLGA^[^ ^37^ ^]^ and PLA^[^ ^38^ ^]^ were taken to be 0.25 and 0.35, respectively. The term *b* accounts for the curvature of the indenter and NP^[^ ^36^ ^]^ and is given by

(2) $$b=\frac{R_{\mathrm{NP}}^{\frac{1}{3}}}{R_{t,\mathrm{NP}}^{\frac{1}{3}}+R_{\mathrm{NP}}^{\frac{1}{3}}}$$

where *R*_NP_ is the radius of the NP, calculated from the height of the NP under test, and *R*_t_ *_,_*_NP_ is calculated as

(3) $$R_{t,\mathrm{NP}}=\frac{R_{t}R_{\mathrm{NP}}}{R_{t}+R_{\mathrm{NP}}}$$

where *R*_t_ is the radius of the tip. Here, *R*_t_ is calculated as the difference between the NP's measured width and its height as the measured width of the NP is represents a convolution of the shapes of the tip and the NP (Figure S9, Supporting Information). The force‐indentation curve was fit from its initial contact point, *d* = 0 nm to *d* = *R*_NP_/10, to avoid substrate effects.^[^ ^39^ ^]^ For each polymer, at least 11 NPs were used and a mean modulus was calculated from the batch.

##### Cell Culture

CD169^+^/CD169^−^ THP‐1 cells have been described previously,^[^ ^4^, ^40^ ^]^ and they were cultured in complete medium containing 10% FBS, 1% penicillin–streptomycin, 2% G418 in RPMI‐1640 medium (Gibco Cell Culture, Thermo Fisher Scientific, Waltham, MA) in a 5% CO_2_ atmosphere at 37 °C. Differentiation of both CD169^+^ and CD169^−^ THP‐1 cells to macrophages were performed by incubation of the cells with 100 × 10^−9^ m PMA (Sigma‐Aldrich, St Louis, MO). CD169^−^ THP‐1 cells were cultured in the same medium without addition of G418.

##### Characterization of GM3‐CD169 Binding in CD169^+^ THP‐1 Cells

5 × 10^5^ CD169^+^ THP‐1 cells (before differentiation) were pelleted after centrifugation (5 min, 270 g). GM3, Gal‐Cer, and without any glycosphingolipids (blank) NPs (10^7^–10^12^ NPs in 0.1 mL of 10% FBS RPMI‐1640) were added to the cell pellets, and incubated at 4 °C for 60 min. Unbound NPs were washed by centrifugation (270 g, 5 min – 2 times), and cells were fixed with 4% PFA (Sigma‐Aldrich, St Louis, MO) for 10 min at room temperature. Following the last washing step, the fluorescence intensity of each sample was measured by flow cytometry using a FACSCalibur instrument (BD Biosciences, San Jose, CA).

For characterization of binding at 37 °C, 5 × 10^11^ GM3, Cal‐Cer, and blank NPs in 0.1 mL 10% FBS RPMI‐1640 were added to the cell pellet, and incubated at 37 °C, 5% CO_2_ for 10 min. Cells were washed, fixed with 4% PFA, and the fluorescence intensity of the samples was determined via flow cytometry. All flow cytometry data were analyzed through Flowing software 2. All fluorescence intensities were background corrected (cells without treatment were used as background), and the calculated difference was used for the analysis.

##### Uptake in Differentiated CD169^+^ THP‐1 Macrophages

CD169^+^ THP‐1 cells after 24 h differentiation with PMA (100 × 10^−9^ m) were lifted using enzyme‐free cell dissociation buffer (Enzyme‐Free Cell Dissociation Solution, Phophate‐Buffered Saline (PBS) based, MilliporeSigma, Burlington, MA). Cells were reseeded at a concentration of 1.25 × 10^5^ cells per well in a 96 well‐plate in complete growth medium, and returned to culture for 24 h. 5 × 10^11^ GM3 NPs in 0.1 mL of 10% FBS RPMI‐1640 were added into each well, and incubated at 37 °C, 5% CO_2_ from 10 min to 6 h. Cells were washed to remove unbound NPs, and lysed with 0.2% Triton‐X 100 (Sigma‐Aldrich, St Louis, MO) in 0.2 N NaOH. The SpectraMax M5 plate reader was used to measure the fluorescence intensity of the cell lysates at excitation and emission wavelengths of 544 and 590 nm. Fluorescence intensities of known concentration of NPs, ranging from 1 × 10^10^ to 5 × 10^12^ NPs mL^−1^, was used to build the calibration curve to determine the number of internalized NPs.

For control experiments, both CD169^+^ and CD169^−^ THP‐1 cells were differentiated with PMA and reseeded in 96‐well plates, as described above. PMA‐differentiated CD169^+^ THP‐1 cells were incubated with either polymer NPs without any membrane or Gal‐Cer NPs. Alternatively, both CD169^+^ and CD169^−^ THP‐1/PMA cells were incubated with GM3 NPs at a concentration of 1 × 10^11^ NPs in 0.1 mL 10% FBS RPMI medium for 10 min at 37 °C, 5% CO_2_. Fluorescence intensity of the cell lysates was determined, as described above.

##### Intracellular Trafficking in CD169^+^ THP‐1 Macrophages

CD169^+^ and CD169^−^ THP‐1 cells were seeded in culture dishes (Cellvis, Mountain View, CA) for differentiation into macrophages (PMA – 48 h). After 10 min incubation of GM3 NPs (5 × 10^11^ NPs in 0.5 mL 10% FBS RPMI‐1640) with cells at 37 °C, 5% CO_2_, cells were washed subsequently with RPMI‐1640 to remove unbound NPs. Following the washing step, cells were incubated in the complete growth medium for 16 h. Cells were incubated with nucleus stain, Hoechst, for 15 min at 37 °C, 5% CO_2_ prior to fixation. Cells were fixed in 4% PFA, permeabilized with 0.2% TWEEN 20, and blocked with 1% BSA in 1 × PBS. To stain CD169, CD9, and LAMP‐1; antihuman CD169 (Sialoadhesin, Siglec‐1, Clone: 7–239), antihuman CD9 (Clone: HI9a), and antihuman CD107a (LAMP‐1, Clone: H4A3) mAbs (BioLegend, San Diego, CA), and Alexa Fluor 647 (Goat antimouse, Clone: Poly4053) conjugated secondary antibody (BioLegend, San Diego, CA) were added successively; a concentration of 1 µg mL^−1^ was used for all antibodies. Multiple washing steps (1 × PBS) were performed after incubation with primary and secondary antibodies. The same procedure was used for lipid‐wrapped polymer NPs with 10 mol% DOPS, without any GM3 (control experiment), and labeled for LAMP‐1.

For control experiments in Figure 5A, polymer NPs without any membrane with CD169^+^ THP‐1 cells, and GM3 NPs with both CD169^+^ and CD169^−^ THP‐1 cells were incubated at 37 °C, 5% CO_2_ for 10 min (same NP concentration). After removing the unbound NPs (without any further 16 h incubation time) cells were fixed with 4% PFA and imaged via confocal microscope. Nucleus stain Hoechst was used to locate the cells. The recorded images were processed by ImageJ to find the NP‐containing area.

##### Image Acquisition and Data Processing

Optical imaging experiments were performed with an Olympus IX71 inverted microscope or Olympus FV1000 scanning confocal microscope. A 10× or 60× oil objective with variable NA (NA = 0.65–1.25) was used to take the images via wide‐filed microscope. Fluorescence imaging was performed under epi‐illumination using appropriate filter sets. Images were recorded with an Andor Ixon^+^ electron multiplying charge coupled device detector. For confocal microscopy, lasers with excitation wavelength of (405, 543, 633 nm) and a 60× (water) objective were used. The recorded images were processed by ImageJ. MCC (M_1_ and M_2_) values were determined via the Coloc 2 analysis plugin (ImageJ).

##### Statistical Analysis

All data are presented as mean ± standard deviation (SD) or mean ± standard error of the mean (SEM), data presentation and sample size for statistical analysis of individual experiments are specified in figure captions. Statistical significance of data was determined using one‐way ANOVA with a subsequent Tukey post‐hoc test as implemented in MATLAB. One asterisk (*) indicates significant differences at *p *≤ 0.05, two asterisks (**) for *p *≤ 0.01, three asterisks (***) for *p *≤ 0.001, and four asterisks (****) for *p *≤ 0.0001. NS was used to demonstrate nonsignificant differences.

##### Ethics Statement

This research has been determined to be exempt by the Institutional Review Board of the Boston University Medical Center since it does not meet the definition of human subjects research, since all human samples were collected in an anonymous fashion and no identifiable private information was collected.

Additional Experimental Section can be found in the Supporting Information.

## Conflict of Interest

S.G. and B.M.R. hold a patent for GM3‐functionalized nanoparticles.

## Author Contributions

B.M.R., B.E. and S.G. planned and designed the experiments. B.E. performed the experiments, including synthesizing the NPs, characterizing the NPs (except TEM and AFM), and all cell studies, and performed the data analysis. N.A. carried out the AFM measurements. N.A. and K.A.B. performed the analysis of the AFM data and the mechanical modeling. X.A. performed TEM imaging, and H. A. provided reagents.

## Supporting information

Supporting Information
