# Supplementary material for: Stiffness of HIV‐1 Mimicking Polymer Nanoparticles Modulates Ganglioside‐Mediated Cellular Uptake and Trafficking
Source: Adv Sci (Weinh). 2020 Jul 29;7(18):2000649. doi: 10.1002/advs.202000649 (PMC7509657; doi:10.1002/advs.202000649)
Supplement: Supplementary file 1 — Supporting Information [file ADVS-7-2000649-s001.pdf]

Copyright WILEY-VCH GmbH, 69469 Weinheim, Germany, 2020.

ADVANCED SCIENCE Open Access

Supporting Information

for *Adv. Sci.,* DOI: 10.1002/advs.202000649

Stiffness of HIV-1 Mimicking Polymer Nanoparticles Modulates Ganglioside-Mediated Cellular Uptake and Trafficking

*Behnaz Eshaghi, Nourin Alsharif, Xingda An, Hisashi Akiyama, Keith A. Brown, Suryaram Gummuluru, and Björn M. Reinhard**

Stiffness of HIV-1 Mimicking Polymer Nanoparticles Modulates Ganglioside-Mediated Cellular Uptake and Trafficking

*Behnaz Eshaghi, Nourin Alsharif, Xingda An, Hisashi Akiyama, Keith A. Brown, Suryaram Gummuluru, and Björn M. Reinhard**

B. Eshaghi, X. An, and Prof. B. M. Reinhard
Department of Chemistry and The Photonics Center
Boston University, Boston, MA 02215, United States
Email: bmr@bu.edu

N. Alsharif and Prof. K. A. Brown
Department of Mechanical Engineering and The Photonics Center
Boston University, Boston, MA 02215, United States

Dr. Hisashi Akiyama and Prof. S. Gummuluru
Department of Microbiology
Boston University School of Medicine, Boston, MA 02118, United States

Supporting Information

Additional Methods

Liposome Preparation: Lipid mixture containing DPPC and cholesterol of a total amount of 10 μmol was dissolved in 1 mL of chloroform in a 25 mL round-bottom flask. The solvent was evaporated, and the samples were dried overnight under vacuum. One mL of 20 mM HEPES buffer was then added to the lipid dry layer. The mixture was then sonicated for 5 min by using a probe sonicator (120 Sonic Dismembrator, Fisher Scientific, Waltham, MA) under argon protection.

Dynamic Light Scattering (DLS) and Zeta Potential Measurements: Size and zeta potential measurements were performed by using Zetasizer Nano ZS90 (Malvern, Worcestershire, UK). For size measurements, NPs were diluted with Milli-Q water. Colloidal stability of NPs were monitored in the cell meida (10% FBS RPMI). The zeta potential of GM3-functionalized polymer NPs were measured in 10 mM NaCl solution.

UV−Vis Spectroscopy: The absorption spectra of polymer NP in Milli-Q water were acquired using Spectronic 200 UV-vis spectrometer (Fisher Scientific, Waltham, MA). Milli-Q water was used for baseline correction. Beer's law was used to calculate the concentration of NPs using the absorption value of fluorescently labeled lipid (Liss Rhod PE) at the wavelength of 570 nm (Lambda max), and the molar absorptivity of $\varepsilon = 73{,}000\ M^{-1}\ cm^{-1}$.

Transmission Electron Microscopy (TEM) Characterization: GM3-presenting polymer and core only NPs drop-cast onto carbon-coated TEM grids and incubated for 20 min before removing the excess solution. Next, samples were stained with 1 % sodium phosphotungstate $Na_3P(W_3O10)_4$ in water (w/v) for 10 sec and excess stain solution was removed. Samples were dried and stored in vacuum before imaging using a Tecnai Osiris transmission electron microscope with a Super-X EDX detection system at an acceleration voltage of 200 kV. TEM, high-resolution TEM, and scanning TEM images and energy dispersive X-ray spectra were obtained.

Biotin-NeutrAvidin Binding Experiment: Rectangular borosilicate capillaries (100 × 2 × 0.1 mm^3, Vitrocom, Mountain Lakes, NJ) were used to perform Biotin-NeutrAvidin binding experiment. Chambers were incubated with 50 µg/ml Bovine Serum Albumin, Biotinylated (BSA-Biotin) (Thermo Fisher Scientific, Waltham, MA) in 0.2 × PBS for 10 min, then 100 µg/mL NeutrAvidin (Thermo Fisher Scientific, Waltham, MA) in 0.2 × PBS was incubated for 10 min, followed by washing with 0.2 × PBS. Next, superblock (SuperBlock (PBS) Blocking Buffer, Thermo Fisher Scientific, Waltham, MA) was incubated in the flow chambers for 30 min. Then, NPs were incubated for 15 min, and unbound NPs were washed by flushing 0.2 × PBS to the flow chambers. Samples were inspected through fluorescence microscopy. The recorded images were processed by ImageJ to calculate the number of NPs bound to the surface.

GM1 Tetramethylbenzidine (TMB)-based Sandwich ELISA Quantification: Relative GM1 concentration were quantified through a TMB-based sandwich ELISA assay in a polylysine-coated 96- well plate. GM1, Gal-Cer, and without any glycosphingolipids (blank) PLGA, PLA, and PLA^{hMW} NPs at a concentration of 10^{12} NPs/mL were incubated in poly-lysine-coated 96-well plate for 1 h. The concentration of NPs was determined before and after loading into the wells. After 3 times washing with the washing buffer (Thermo Fisher Scientific, Waltham, MA), 200 µL rabbit polyclonal anti-GM1 antibody (ab23943, abcam, Cambridge, MA) (dilution 1:1000) in 0.1 mg/mL BSA in 1 × PBS was added into each well and incubated at room temperature for 2 h. Next, each well washed three times to remove the excess antibodies. Following the washing step, 200 µL goat anti-rabbit IgG HRP-conjugated antibody (ab205718, abcam, Cambridge, MA) 10 ng/mL in 0.1 mg/mL in 1 × PBS was added into each well and incubated at room temperature for 1 h. After three times washing, 100 µL TMB solution (0.5 mM) (Thermo Fisher Scientific, Waltham, MA) was added into each well and incubated for 10 min at room temperature before adding the stop solution (Thermo Fisher Scientific, Waltham,

MA). The SpectraMax M5 plate reader (WVR, Radnor Corporate Center, Radnor, PA) was used to measure the absorbance of samples at excitation wavelength of 450 nm.

Optical Colocalization of Polymer Core and Membrane of GM3-functionalized Polymer NPs: Rectangular borosilicate capillaries (100 × 2 × 0.1 mm^3, Vitrocom, Mountain Lakes, NJ) were used to perform colocalization experiment. Chambers were incubated with 0.1% poly-L-lysine for 10 min. Then, GM3 NPs diluted in 0.2 × PBS were incubated for 10 min, and unbound NPs were washed by flushing 0.2 × PBS to the flow chambers. Samples were inspected through fluorescence microscopy. The recorded images were processed by ImageJ to process and overlay the fluorescence images of the core and the membrane. The images were corrected for the drift.

Optical microscopy for Stability of the Membrane in Cell-lysate: Rectangular borosilicate capillaries (100 × 2 × 0.1 mm^3) were incubated with 0.1% poly-L-lysine for 10 min. Then, GM3-functionalzied PLGA NPs in 0.2 × PBS were incubated for 10 min, and unbound NPs were washed by flushing 0.2 × PBS into the flow chambers. Next, cell lysate of $CD169^+$ THP-1 cells were flushed into the chamber. The same field of view was inspected through fluorescence microscopy for up to 5 days. The recorded images were processed by ImageJ to overlay the fluorescence images immediately after addition of the cell-lysates and after different incubation times. The images were corrected for drift.

Cell Viability 3-(4, 5-dimethylthiazol-2-yl)-2, 5-diphenylterazolium bromide (MTT) Assays: Cell viability was quantified through MTT assays. After 24 h differentiation with PMA (100 nM) $CD169^+$ THP-1 cells were lifted off using enzyme free cell dissociation buffer (Enzyme-Free Cell Dissociation Solution PBS based, MilliporeSigma, Burlington, MA). Then, cells were counted and reseeded at a concentration of 1.25×10^5 cells per well in a 96 well-plate using the complete growth medium. Cells were incubated with various concentrations of GM3-presenting NPs (1×10^{11}, 1×10^{12}, and 5×10^{12} NPs/mL) for 10 min, and for some conditions, after removing the NPs, cells were maintained in the full media for 16 h. Then cells were

subsequently washed and incubated with 100 μL of RPMI containing 10% MTT solution (5mg/mL) for 3 h at 37°C. After incubation, the MTT solution was removed, and 100 μL of a DMSO and ethanol solution (1:1 ratio) was added to each well and mixed thoroughly. Absorbance was measured at 570 nm using a SpectraMax M5 plate reader (WVR, Radnor Corporate Center, Radnor, PA).

Differential Scanning Calorimetry (DSC) Measurements: Polymer NPs (with and without membrane) were concentrated by a factor of 3. To characterize thermal behavior of polymer NPs (with and without membrane) and liposomes, 30 μL of solution was transferred into an aluminum pan (Thermal Support, Hayesville, NC) and sealed. For the dried NPs, 30 μL of the NPs solution was transferred to the DSC pan and air dried for 4 h, following 4 h further drying at 35 °C before sealing the pans. Thermograms from 25 °C to 75 °C, at a heating rate of 5 °C/min were recorded by using a Mettler Toledo Polymer DSC R (Mettler-Toledo, Columbus, OH). For polymer powder measurements, samples were sealed in the aluminum pans, and thermograms were recorded under the same experimental conditions. DSC software was used to determine the glass transition and phase transition temperatures.

Monocytes Derived Macrophages (MDMs) Culturing and Staining: Human MDMs were derived from positively isolated $CD14^+$ peripheral blood monocytes by culturing in RPMI-1640 containing 10% heat-inactivated human AB serum (Sigma-Aldrich) and recombinant human macrophage colony stimulating factor (M-CSF) (20 ng/mL; PeproTech) for 5–6 days. Blood products were purchased from NY Biologics.

After 10 min incubation of GM3-presenting PLA^{hMW} NPs (5×10^{11} NPs in 0.5 mL 10% FBS RPMI-1640) with cells at 37 °C, 5% CO_2, cells were washed with RPMI-1640 to remove the unbound NPs. Following the washing step, cells were incubated in the complete growth medium for 16 h. Same staining protocol as described in the methods section was repeated to stain for nucleus, CD169, CD9, and LAMP-1. Next, samples were imaged via confocal microscope.

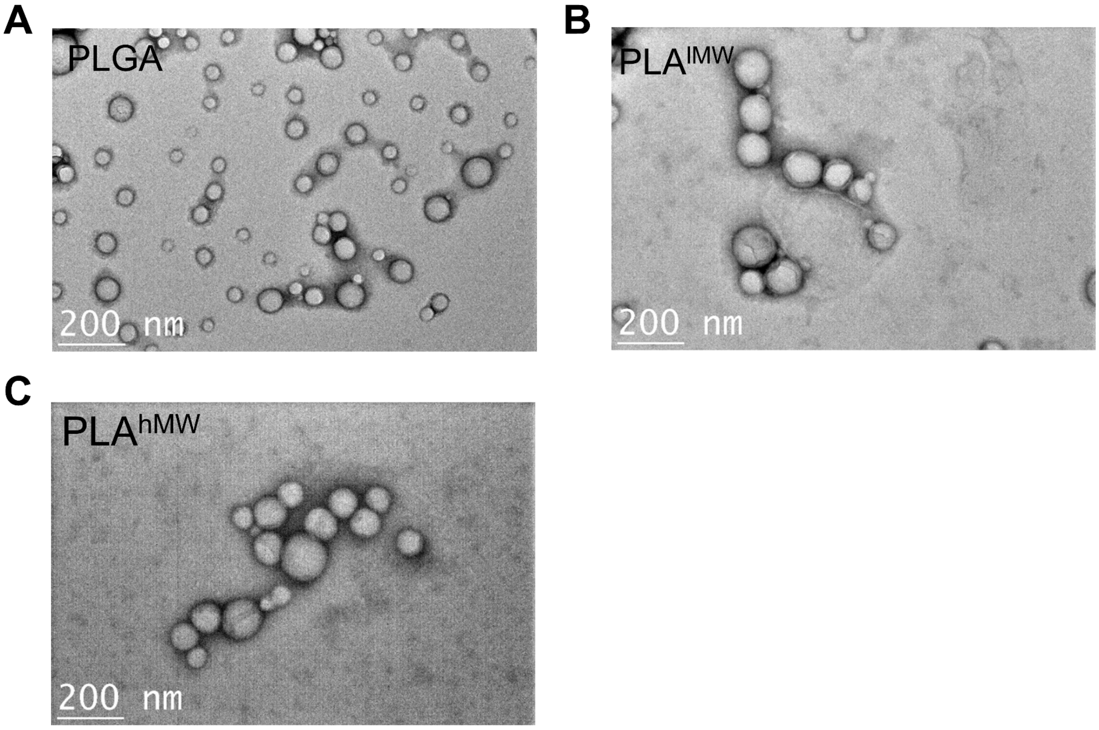

Figure S1. High-resolution TEM images of GM3-presenting lipid-wrapped PLGA, PLA^{lMW}, and PLA^{hMW} NPs.

The TEM images of (**A**) PLGA, (**B**) PLA^{lMW}, and (**C**) PLA^{hMW} NPs. All NPs were treated with 1% (w/v) sodium phosphotungstate $Na_3P(W_3O_{10})_4$. Any “clustering” in the TEM images is a by-product of the sample preparation on the TEM grids.

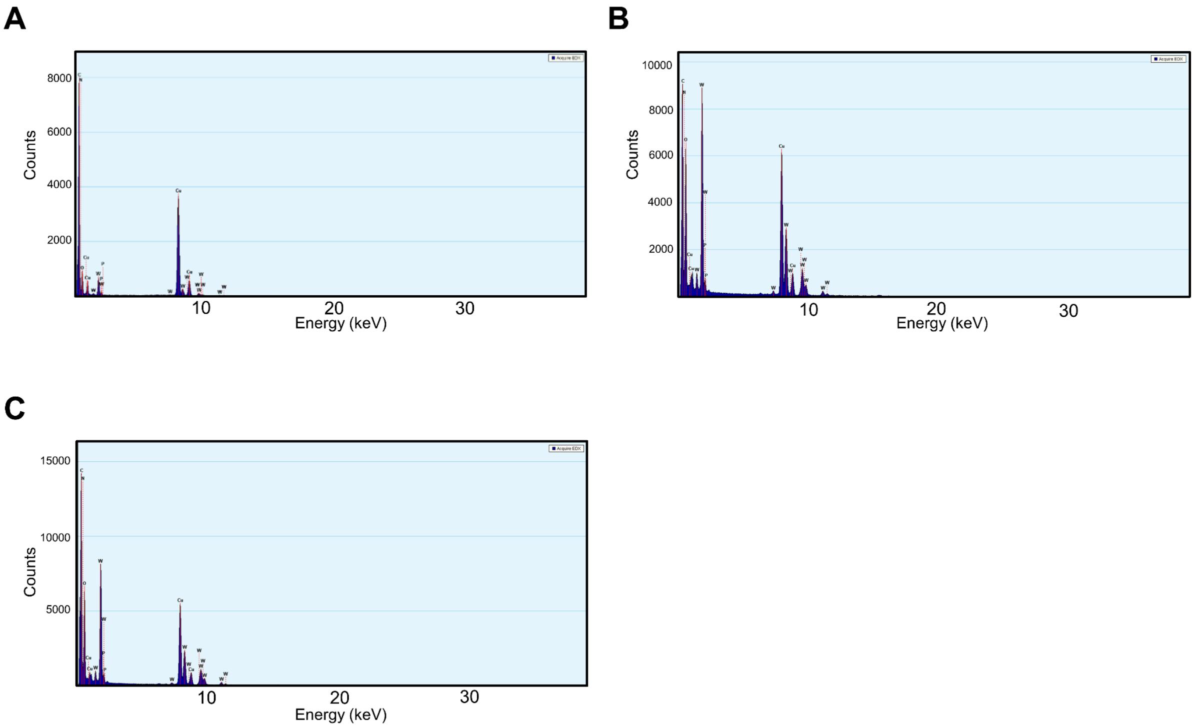

Figure S2. Energy dispersive X-Ray (EDX) spectra of GM3-preseting PLGA, PLA^{lMW}, and PLA^{hMW} NPs imaged with TEM.

EDX spectra of (**A**) PLGA, (**B**) PLA^{lMW}, and (**C**) PLA^{hMW} GM3-presenting lipid-wrapped NPs.

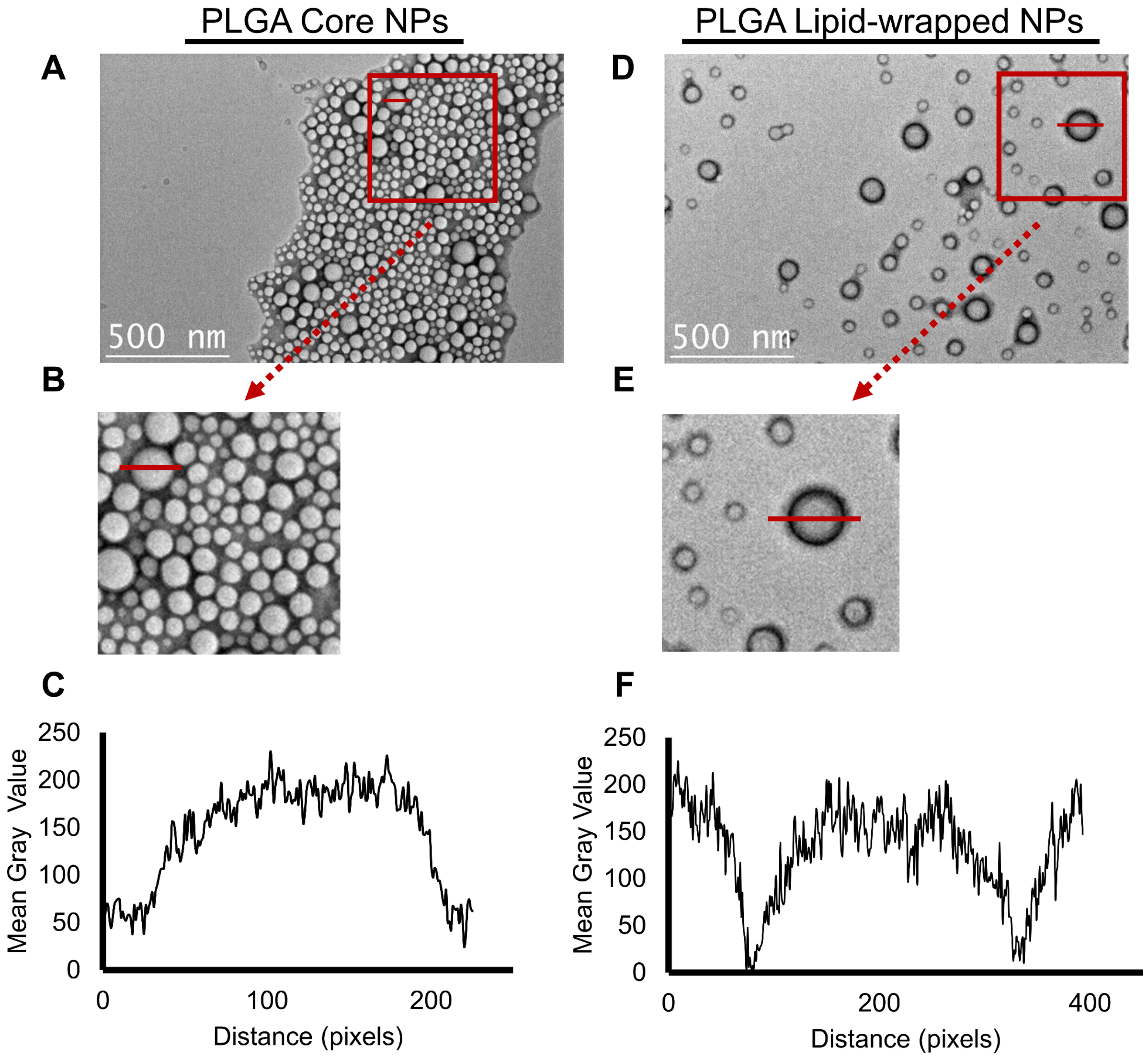

Figure S3. TEM characterization of GM3-functionalized lipid-wrapped PLGA NPs and PLGA core NPs (no membrane).

(**A**) High-resolution TEM of PLGA core NP (without membrane), (**B**) magnified view of the selected area in (A). (**C**) Mean gray value of pixels along the red line for the representative NP shown in (A) and (B) (determined with ImageJ). (**D**) High-resolution TEM of GM3-presenting lipid-wrapped PLGA NPs, (**E**) magnified view of the selected area in (D). (**F**) Mean gray value of pixels along the red line for the representative NP shown in (D) and (E) (determined with ImageJ). Both samples were stained with 1% (w/v) sodium phosphotungstate $Na_3P(W_3O_{10})_4$. The membrane-wrapped NPs show a stronger accumulation of $Na_3P(W_3O_{10})_4$ at the periphery than the control NPs without membrane.

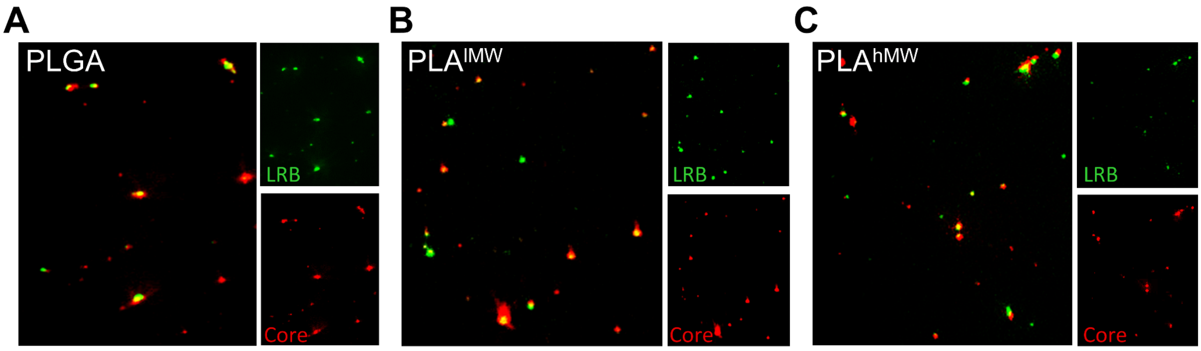

Figure S4. Optical colocalization of the core and membrane of GM3-functionalized PLGA, PLA^{lMW}, and PLA^{hMW} NPs.

Hydrophobic dye (coumarin) incorporated in the core and the lipid dye (Liss Rhod PE) in the membrane of the NPs generate fluorescence signal. The overlay (yellow) and the individual fluorescence images of core (red) and membrane (green) are shown in (**A**) PLGA, (**B**) PLA^{lMW}, and (**C**) PLA^{hMW} NPs.

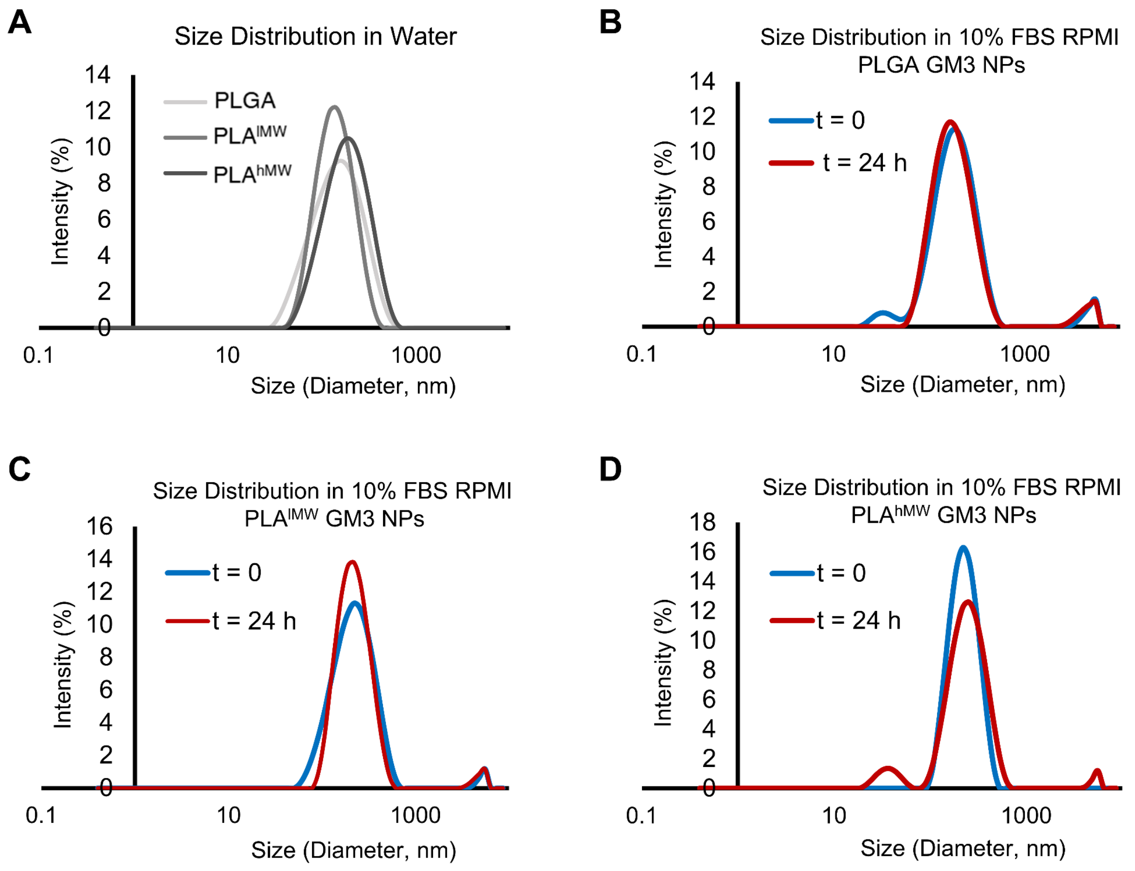

Figure S5. Hydrodynamic size of the GM3-presenting PLGA, PLA^{lMW}, and PLA^{hMW} NPs by DLS in water and 10% FBS RPMI.

(**A**) Intensity statistics of size measurements of PLGA, PLA^{lMW} and PLA^{hMW} NPs in water. (**B-D**) Intensity statistics of size measurements of PLGA (**B**), PLA^{lMW} (**C**), and PLA^{hMW} (**D**) GM3-presenting NPs in the 10% FBS RPMI immediately and 24 h after incubation.

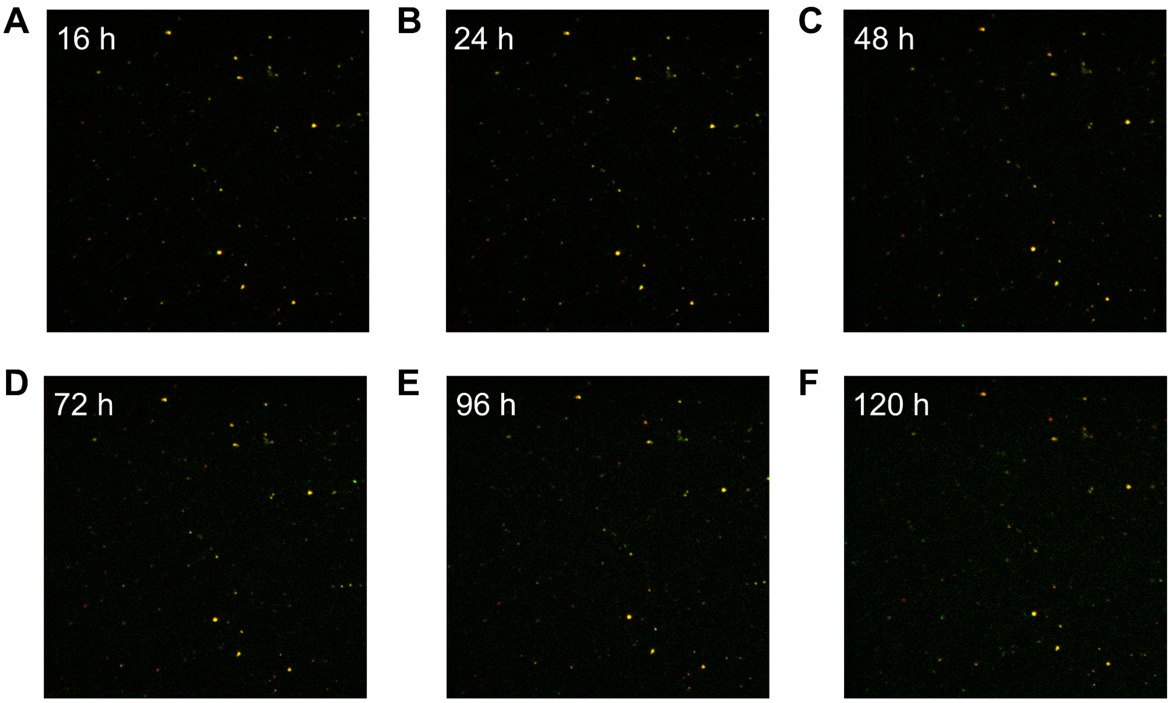

Figure S6. Stability of the GM3-functionalized PLGA NPs in CD169$^+$ THP-1 cell lysates. Optical microscopy of surface-immobilized fluorescently labeled lipid-wrapped GM3-included PLGA NPs with cell lysates. The overlay (yellow) fluorescence images of immediately after addition of cell-lysates into flow-chamber (red) and after different incubation times (green) are shown in (**A**) after 16 h, (**B**) after 24 h, (**C**) after 48h, (**D**) after 72 h, (**E**) after 96 h, and (**F**) after 120 h. PLGA was chosen for the stability test as it has the fastest degradation rate among of all the investigated polymers.

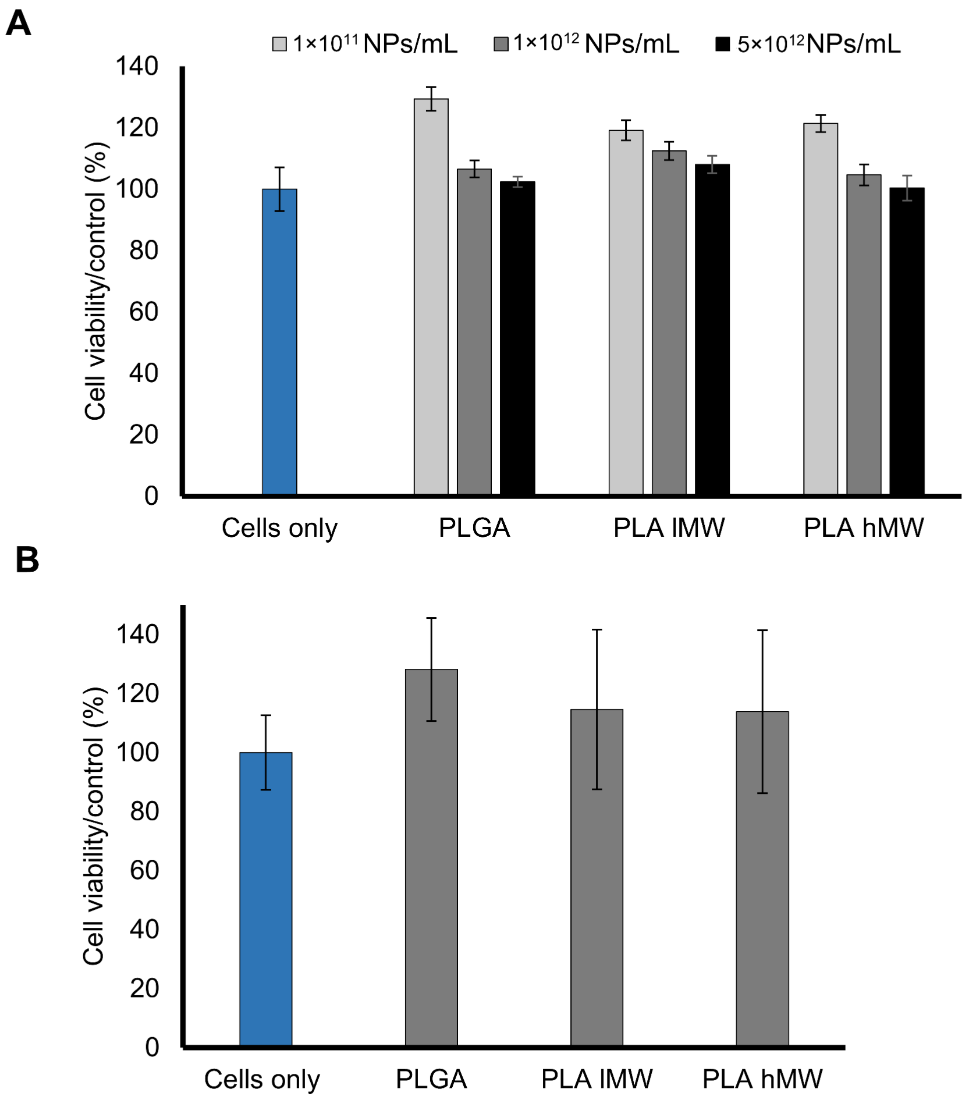

Figure S7. MTT cell viability assay for GM3-functionalized PLGA, PLA^{lMW}, and PLA^{hMW} NPs in $CD169^{+}$ THP-1 after differentiation (macrophages).

Cell viability was measured (**A**) after 10 min incubation of NPs with a concentration of 1×10^{11}, 1×10^{12}, and 5×10^{12} NPs/mL and (**B**) after 10 min incubation of NPs with the concentration of 1×10^{12} NPs/mL and subsequent chase of 16 h after removal of the NPs.

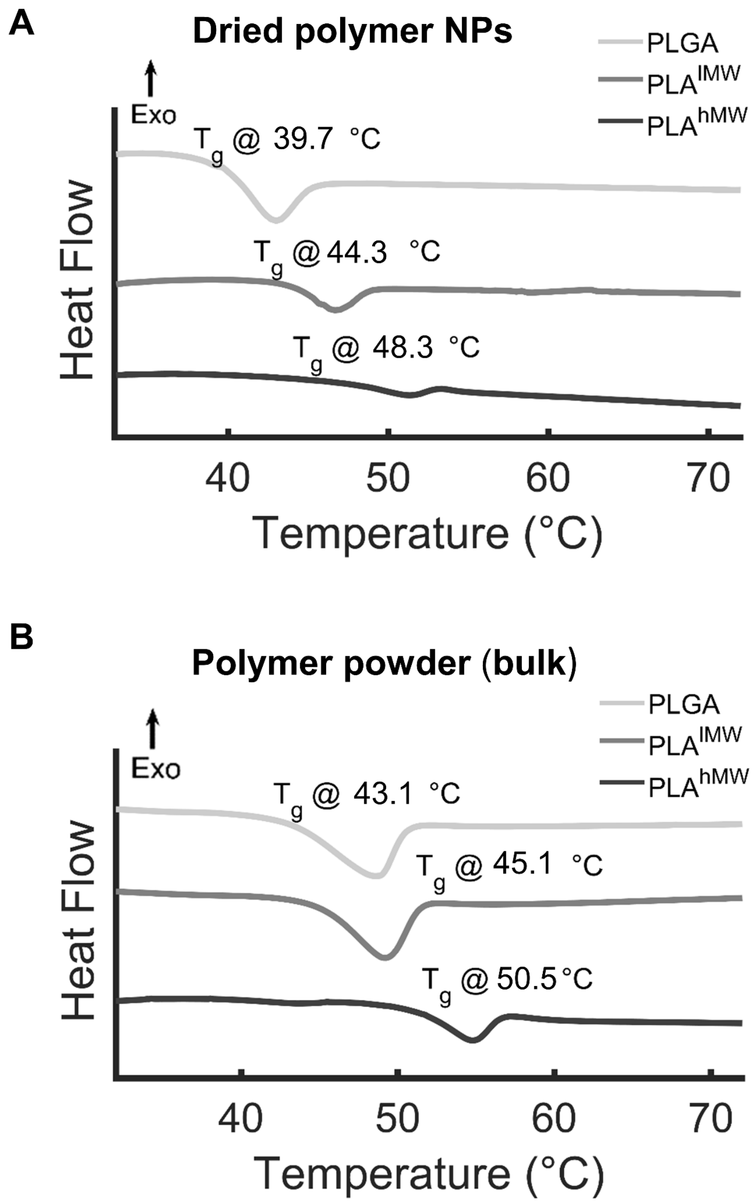

Figure S8. DSC thermograms of dried NPs and powder of PLGA, PLA^{lMW}, and PLA^{hMW}. (**A**) DSC plot of dried PLGA, PLA^{lMW}, and PLA^{hMW} NPs with glass transition temperature (T_g) in the range of 39 – 49 °C. The shape of the phase transitions were previously attributed to the coexistence of glass transition and relaxation endotherms (reference 17). (**B**) DSC plots of powder (bulk) of PLGA, PLA^{lMW}, and PLA^{hMW}. DSC software was used to determine the glass transition temperature and the onset values are reported on the plots.

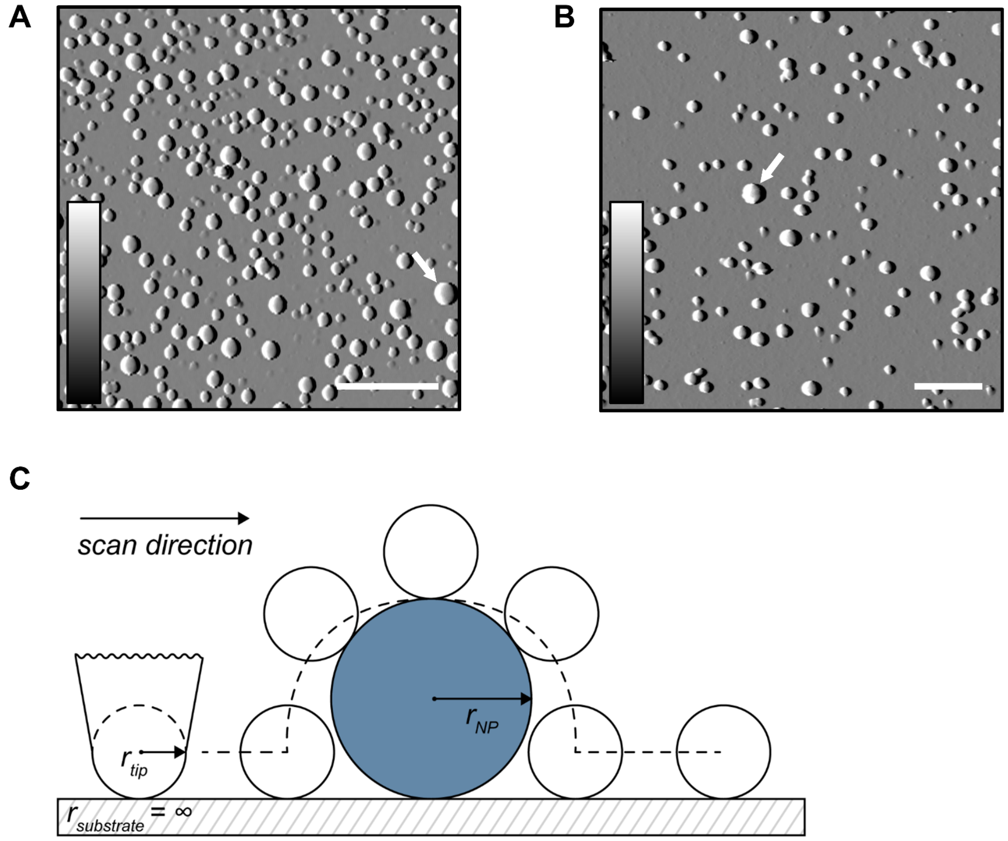

Figure S9. Characterization of the mechanical properties of polymers NPs by atomic force microscopy (AFM).

(A) Typical AFM tapping mode amplitude map of a 2 × 2 μm^2 scan of PLGA NPs, dried at room temperature, scale bar = 500 nm, with amplitude colorbar range = 75.5 – 98.5 nm, tallest nanoparticle height is measured from height channel at 75 nm, denoted by white arrow. (B) Typical AFM tapping mode amplitude map of a 3 × 3 μm^2 scan of PLA^{hMW} NPs, dried at room temperature, scale bar = 500 nm, with amplitude colorbar range = 62 – 87 nm, tallest nanoparticle height is measured from height channel at 127 nm, denoted by white arrow. (C) Schematic of AFM scanning, showing approximate proportions of tip radius and NP size, necessitating the need for the modified Hertzian contact model. Dashed line indicates the measured particle size by way of tip convolution.

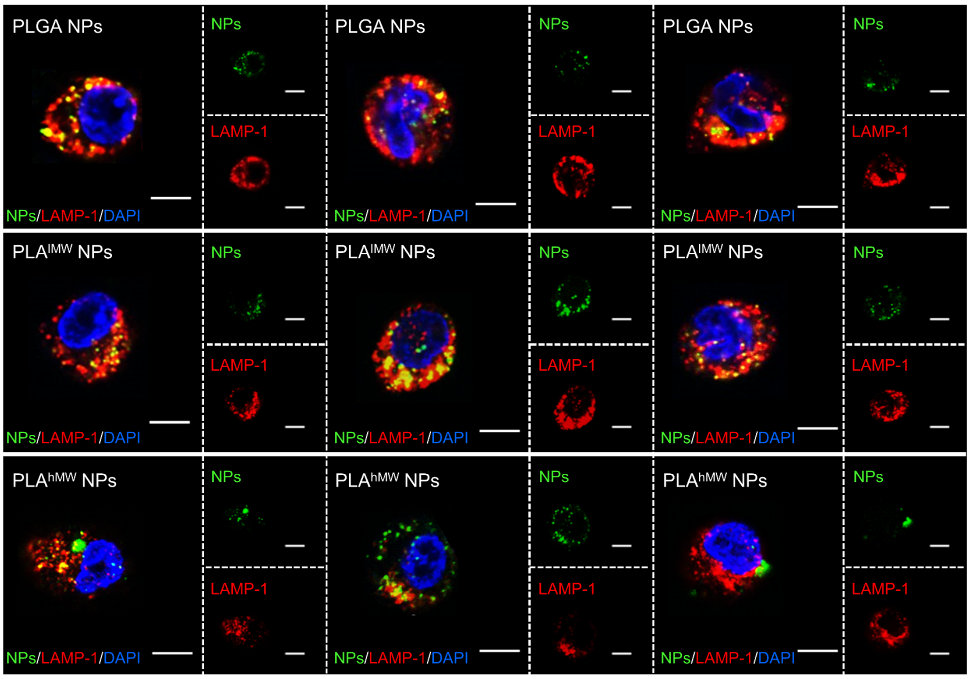

Figure S10. Characterizing the intracellular fate of GM3-presenting PLGA, PLA^{lMW}, and PLA^{hMW} NPs in LAMP-1 immunolabeled CD169-expressing macrophages.

Confocal fluorescent images of CD169-expressing macrophages (CD169^{+} THP-1 cells after differentiation) after 10 min incubation with GM3-presenting PLGA, PLA^{lMW}, and PLA^{hMW} NPs and subsequent chase of 16 h and stained for LAMP-1, scale bar = 5 µm.

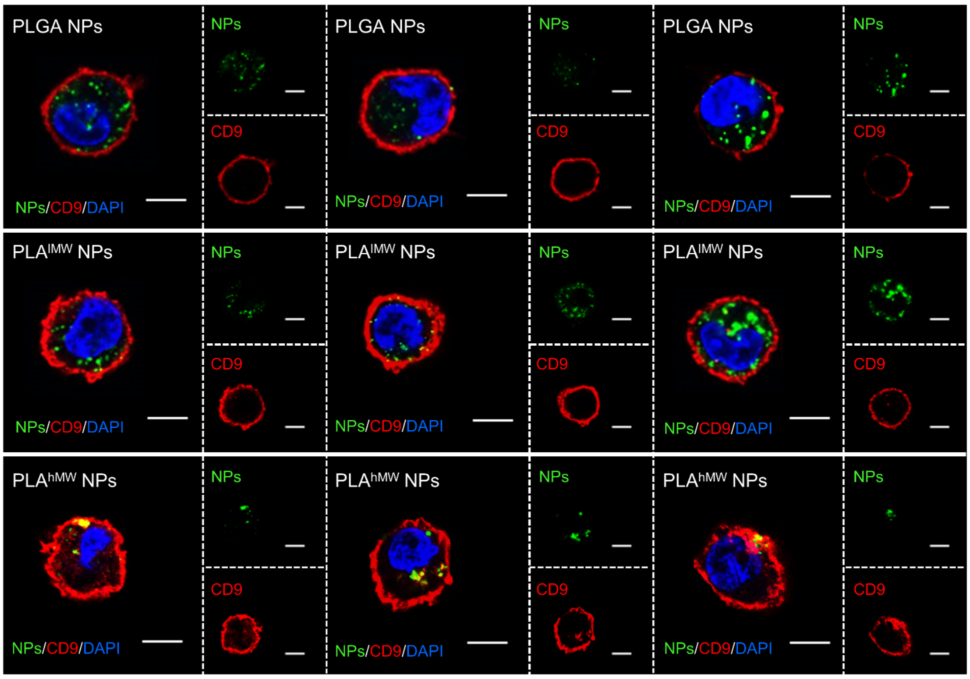

Figure S11. Mapping the intracellular fate of GM3-presenting PLGA, PLA^{lMW}, and PLA^{hMW} NPs in CD9 immunolabeled CD169-expressing macrophages.

Confocal fluorescent images of CD169-expressing macrophages (CD169^{+} THP-1 cells after differentiation) after 10 min incubation with GM3-presenting PLGA, PLA^{lMW}, and PLA^{hMW} NPs and subsequent chase of 16 h and stained for CD9, scale bar = 5 µm.

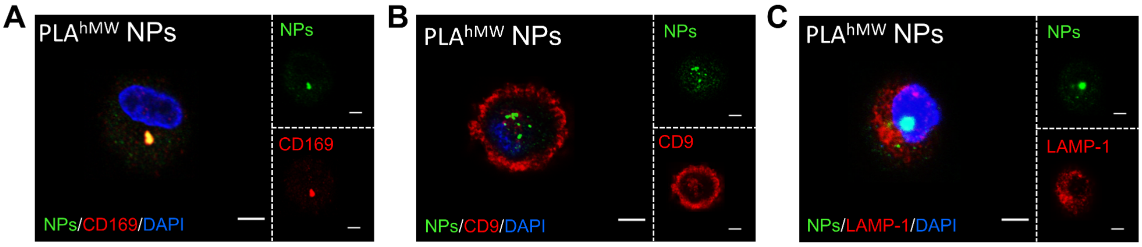

Figure S12. Mapping the intracellular fate of GM3-presenting PLA^{hMW} NPs in human monocyte derived macrophages (MDMs) after 10 min of incubation with NPs and subsequent chase of 16 h and staining for CD169, CD9, and LAMP-1.

(**A-C**) Confocal sections of macrophages containing fluorescent PLA^{hMW} NPs and stained for (**A**) CD169, (**B**) CD9, and (**C**) LAMP-1 (scale bar = 5 µm).

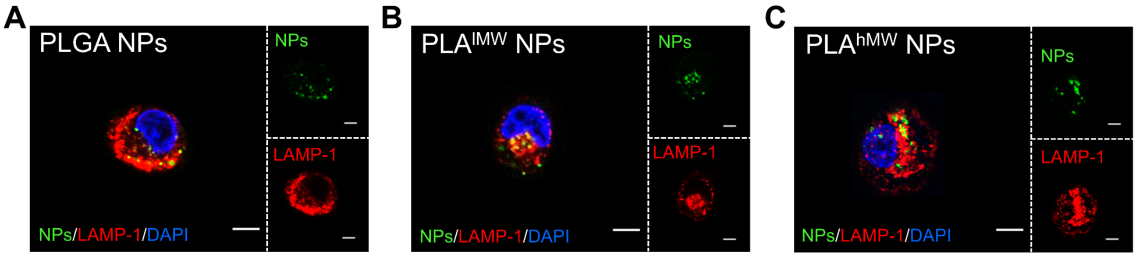

Figure S13. Characterizing the intracellular fate of PLGA, PLA^{lMW}, and PLA^{hMW} NPs with 10 mol% DOPS in their membrane.

(**A-C**) Confocal fluorescent images of CD169-expressing macrophages (CD169^{+} THP-1 cells after differentiation) incubated with DOPS-containing PLGA (**A**), PLA^{lMW} (**B**) and PLA^{hMW} (**C**) NPs for 10 min and and subsequent chase of 16 h and stained for LAMP-1, scale bar = 5 μm.
